# TAFFYS: An Integrated Tool for Comprehensive Analysis of Genomic Aberrations in Tumor Samples

**DOI:** 10.1371/journal.pone.0129835

**Published:** 2015-06-25

**Authors:** Yuanning Liu, Ao Li, Huanqing Feng, Minghui Wang

**Affiliations:** 1 School of Information Science and Technology, University of Science and Technology of China, Hefei, AH230027, China; 2 Research centres for Biomedical Engineering, University of Science and Technology of China, Hefei, AH230027, China; Sapporo Medical University, JAPAN

## Abstract

**Background:**

Tumor single nucleotide polymorphism (SNP) array is a common platform for investigating the cancer genomic aberration and the functionally important altered genes. Original SNP array signals are usually corrupted by noise, and need to be de-convoluted into absolute copy number profile by analytical methods. Unfortunately, in contrast with the popularity of tumor Affymetrix SNP array, the methods that are specifically designed for this platform are still limited. The complicated characteristics of noise in signals is one of the difficulties for dissecting tumor Affymetrix SNP array data, as they inevitably blur the distinction between aberrations and create an obstacle for the copy number aberration (CNA) identification.

**Results:**

We propose a tool named TAFFYS for comprehensive analysis of tumor Affymetrix SNP array data. TAFFYS introduce a wavelet-based de-noising approach and copy number-specific signal variance model for suppressing and modelling the noise in signals. Then a hidden Markov model is employed for copy number inference. Finally, by using the absolute copy number profile, statistical significance of each aberration region is calculated in term of different aberration types, including amplification, deletion and loss of heterozygosity (LOH). The result shows that copy number specific-variance model and wavelet de-noising algorithm fits well with the Affymetrix SNP array signals, leading to more accurate estimation for diluted tumor sample (even with only 30% of cancer cells) than other existed methods. Results of examinations also demonstrate a good compatibility and extensibility for different Affymetrix SNP array platforms. Application on the 35 breast tumor samples shows that TAFFYS can automatically dissect the tumor samples and reveal statistically significant aberration regions where cancer-related genes locate.

**Conclusions:**

TAFFYS provide an efficient and convenient tool for identifying the copy number alteration and allelic imbalance and assessing the recurrent aberrations for the tumor Affymetrix SNP array data.

## Background

Accurate detection of cancer genomic aberrations can greatly facilitate field of cancer genome study and personalized clinical therapeutic treatment [1]. Advances in high-throughput genomic technologies, including single nucleotide polymorphism genotyping microarray (SNP array) [2] and next-generation sequencing (NGS) [3], provide powerful tools to pinpoint genomic aberrations in cancer cells [4]. SNP array represents a high quality and cost-efficient platform with advantage for simultaneous detection of both copy number aberration and allelic imbalance, and has been widely adopted in cancer related studies [5]. Along with the accumulation of tumor samples, a convenient and efficient tool that focuses on aberration analysis will be helpful in genome studies.

**Fig 1:**
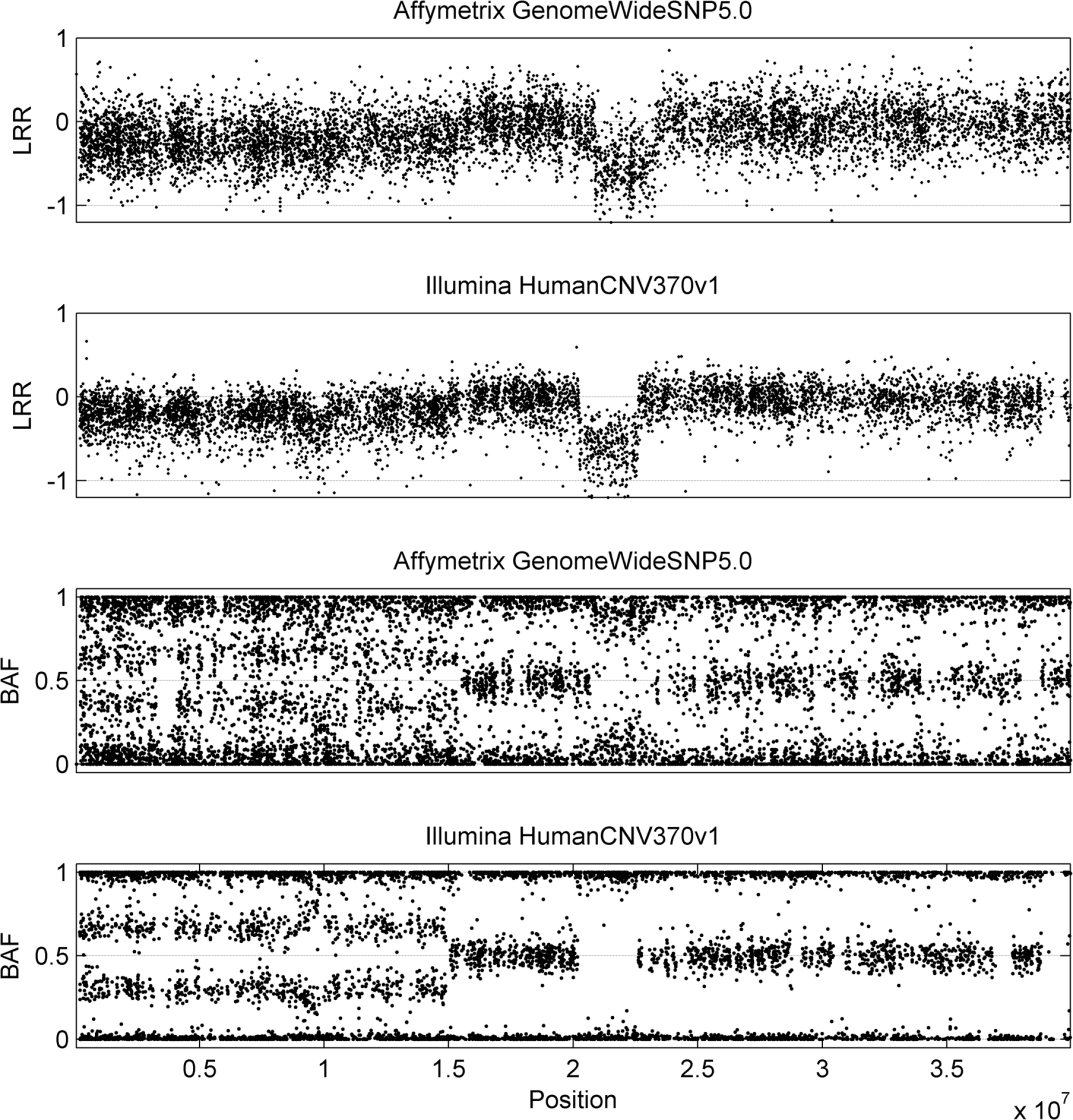
Comparison of genotyping signals between Affymetrix and Illumina platform. The Log R Ratio (LRR) and B Allele Frequency (BAF) signals are illustrated for breast tumor sample H1395, which is analyzed by both Affymetrix GenomeWide 5.0 and Illumina HumanCNV370v1 platform.

Suppose the genotype of one SNP can be denoted with two alleles ‘A’ and ‘B’, and SNP array signals contain two measurements for each SNP: Log R Ratio (LRR) and B Allele Frequency (BAF), which denote the relative total copy number and the fraction of B allele, respectively [2,6]. By de-ciphering the LRR and BAF signals, the genotype can be ascertained. For example, the diploid genotype ‘AB’ normally produces the LRR signal around 0, and the BAF signal around 0.5. With the gain of copy number, the LRR signal is normally elevated and BAF signal changes according to the fraction of B allele in altered genotype. At present, a large number of analytical methods have been proposed to de-convolute absolute copy number profile from noisy SNP array signals [7–16], but only few of them are designed for Affymetrix SNP array. One difference between Affymetrix and other platforms, e.g. Illumina platform, is that signals from the former are more complicated, as shown in Fig 1. Totally 6,903 probes from Illumina HumanCNV 370k platform and 8,245 probes from Affymetrix GenomeWideSNP 5.0 platform are shown for comparison. Specifically, compared with Illumina platform, the signals from Affymetrix are apparently noisy with very large and non-uniform variances for different aberration regions, indicating low signal-to-noise ratio (SNR) and aberration-related signal variance. As a result, this Affymetrix-specific noise inevitably blurs distinction between aberrations and creates an obstacle for the copy number alteration (CNA) identification. Therefore, efficient methods for Affymetrix SNP arrays are needed for systematic analysis of vast amounts of tumor samples that are readily available, such as public database Gene Expression Omnibus (GEO). So far, several methods have been introduced for this purpose [13,14,16], but they still have some drawbacks. For example, OncoSNP [16] and ASCAT [14], which are initially designed for Illumina platform, have been further extended into Affymetrix platform for aberration detection. While these expansions adjust parameters for Affymetrix signals, they do not adequately address the aforementioned noise problems. Another approach named TAPS [13] is proposed for Affymetrix SNP array, which simultaneously takes tumor aneuploidy and intra-heterogeneity into consideration. To overcome the problems caused by poor signal quality and guarantee the reliability of interpretation, this method requires manual inspection to assign parameters for each sample. Considering the dependence on manual intervention, it may not be convenient for the studies with a large number of tumor samples.

Another application of Affymetrix SNP array lies in the field of recurrent aberration identification [17,18]. Compared with non-recurrent aberration which is assumed to be randomly distributed cross the genome, recurrent aberration has growth advantage in cancer cell population, and is positively selected during the evolution of the cancer. Therefore, the study on recurrent aberration might provide a good insight about the progression of cancer. By using multiple tumor samples, the statistical significance of genomic aberration can be quantitatively calibrated and thus facilitate detection of recurrent aberration. Previous study provides an efficient framework for statistical significance assessment [17]. However, this framework can be further renovated by using absolute copy number profile provided by TAFFYS, and potential advantages will be achieved. For example, the utility of absolute copy number can efficiently avoid the bias caused by normal cell contamination, as well as the noise in original signals. Besides, this strategy can also extend the analysis from copy number alteration to allelic imbalance, e.g. LOH, which has also been proved to be associated with cancer development [14].

In this study, we present an efficient bioinformatic tool devoted to comprehensive analysis of genomic aberrations from tumor Affymetrix SNP array data (TAFFYS). By carefully investigating the signal distributions of Affymetrix SNP array, we propose a wavelet-based de-noising approach and a copy number-specific variance model for suppressing and modelling the noise in original signals. Processed signals are then quantitatively modelled for genomic aberration identification. Finally, based on the results of copy number inference, a significance test is performed to discover recurrent and functionally important aberrations that play an important role in tumorigenesis and tumor progression.

## Methods

### Overview

TAFFYS offers an integrated solution for Affymetrix tumor SNP array data analysis and the pipeline is shown in S1 Fig. First, Affymetrix CEL file is pre-processed to extract genotyping signals. The PennCNV-affy [15] built-in module transforms the normalized signals into LRR and BAF, and then a wavelet de-noising approach is applied to suppressing noise of LRR signals. Based on the statistical distributions of the LRR and BAF signals, TAFFYS adopts a hidden Markov model (HMM) and expectation maximization (EM) algorithm for identification of genomic aberration and tumor genotype, in which critical issues including signal variances, normal cell contamination [10–14,16], LRR baseline shift [9,10,14,16] and GC content bias [19] are parameterized and estimated. In addition, for multiple tumor samples, TAFFYS provides a permutation-based approach by using the absolute copy number profile to evaluate the statistical significance of aberration in cancer genome. Details see section *Software introduction* in Supplementary material.

### Statistical distributions of Affymetrix genotyping signals

#### BAF signals

**Fig 2:**
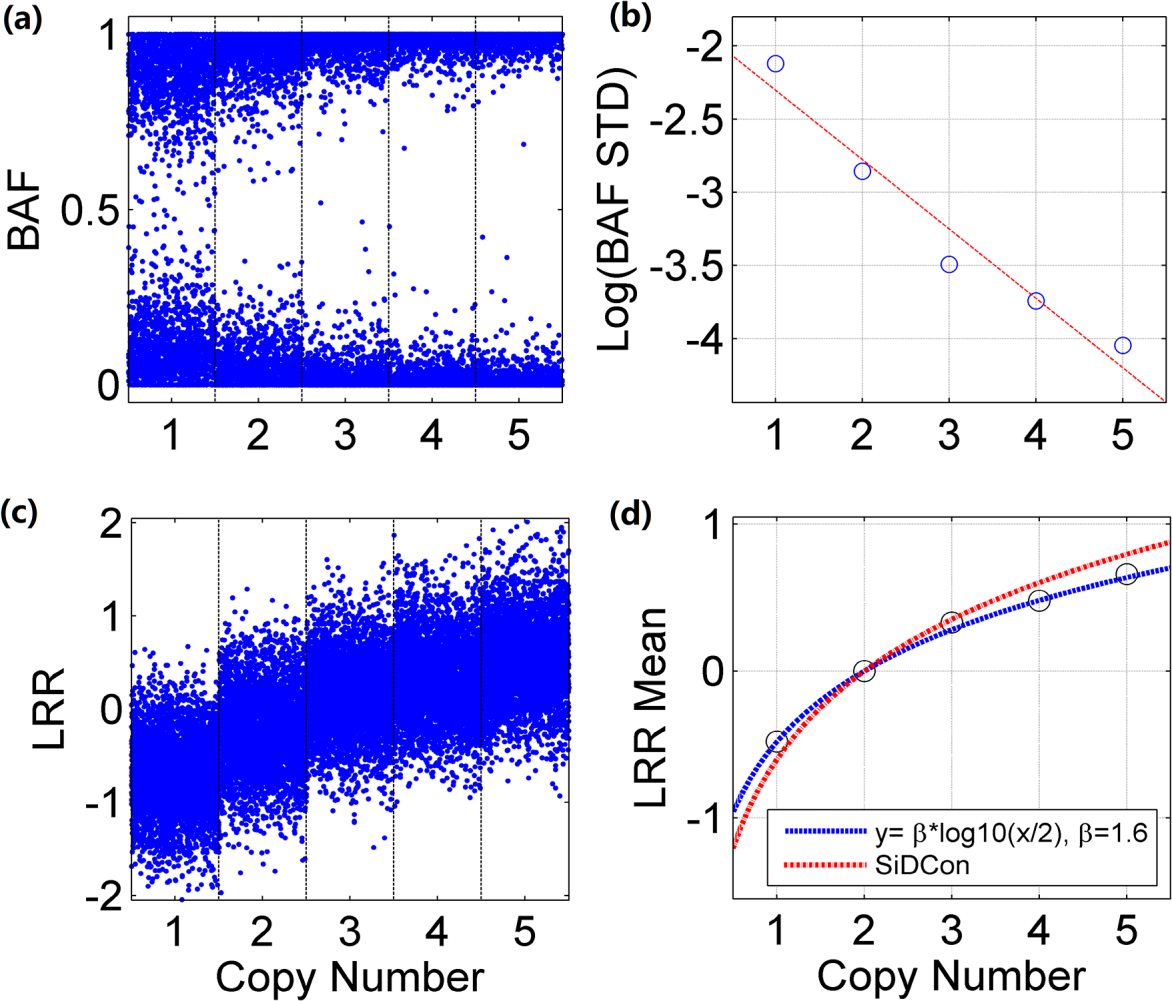
Comparison of LRR and BAF signals with respect to different copy numbers. The Log R Ratio (LRR) and B Allele Frequency (BAF) signals are illustrated with respect to different copy numbers. (a) Statistical distributions of BAF signals from homozygosity SNPs are shown with copy number from 1 to 5. (b) Comparison of BAF signal variances shows that the relationship of variance and copy number can be approximated by a log-linear function. (c) Statistical distributions of LRR signals are illustrated with copy number ranging from 1 to 5. (d) Comparison of real mean values of LRR and theoretical values calculated by two formulas for different copy numbers.

As the first step, we investigate BAF signals for Affymetrix platform, and Fig 2A illustrates the distributions of BAF signals with respect to different copy numbers for a lung cancer cell-line sample H1395 (which is available from GEO website with accession number [GEO: GSE26302]). The variance of BAF signals associated with homozygous tumor genotypes, e.g. ‘B’ and ‘AAAA’, dramatically rises when tumor copy number decreases. Further examination (Fig 2B) shows this relationship can be approximated by a log-linear function:

$$log\left(\sigma_{n_{t}}^{Bhom}\right)-log\left(\sigma_{2}^{Bhom}\right)=K\left(n_{t}-2\right)$$ (1)

here $\sigma_{2}^{Bhom}$ is the standard deviation (STD) of BAF signals for diploid tumor genotypes, and $\sigma_{n_{t}}^{Bhom}$ is the STD of BAF signals associated with copy number *n*_*t*_. The slope of the fitted line, *K*, represents an increment coefficient against tumor copy number *n*_*t*_. This equation can be further written as:

$$\sigma_{n_{t}}^{Bhom}=\sigma_{2}^{Bhom}e^{K(n_{t}-2)}$$ (2)

Eq (2) will be used in HMM for detection of genomic aberrations (see Section *Emission probability function*). In addition, to reduce computational complexity, BAF signals are upward mirrored along the 0.5 axis in TAFFYS.

#### LRR signals

Next, the statistical distributions of LRR signals for sample H1395 are investigated and shown in Fig 2C. The variances of Affymetrix LRR signals are consistent (~0.17) for different copy numbers and usually are about 3 times larger than these of Illumina platform (~0.04) [9,10]. Such high noise creates an obstacle for precisely detecting genomic aberration. To address this issue, a de-noising procedure is adopted in TAFFYS to increase the SNR of LRR signals (see next section). At the same time, Fig 2D shows the mean of LRR signals for Affymetrix platform does not fit to a previously proposed empirical formula for Illumina platform [5], and therefore we propose a modified formula for Affymetrix platform by adding a contraction coefficient *β*:

$$mean(l)=\beta \;*\;{log}_{10}(\frac{n_{t}}{2})$$ (3)

here *l* represents LRR signals associated with copy number *n*_*t*_. As illustrated in Fig 2D, by selecting an appropriate *β* Eq (3) can accurately delineate the statistical behaviour of LRR signals when copy number alters.

#### Wavelet-based signal de-noising

As discussed above, the issue of low SNR in Affymetrix LRR signals greatly hampers interpretation of tumor SNP array data and therefore need be addressed before further signals modelling and analysis. Generally, LRR signals represent mixtures of block signals with additive white Gaussian noise, featured by distinct aberration regions and sharp changes of LRR signals at the breakpoint of two adjacent regions. The underlying idea behind wavelet-based de-noising is to treat signal as a linear combination of wavelets. By decompositing the raw signals, the reflections of noise and indicative signal can be obtained. Then the noise part is discarded for reconstructing the clean signal. To suppress the noise and meanwhile recovery the original LRR signals, TAFFYS adopts a de-noising pre-processing procedure based on wavelet, which was suggested by Hsu [20], and this process mainly contains three steps:

The decomposition of wavelet signal: Firstly, the wavelet transform decomposes each level of signals with two complementary high- and low-pass filters determined by specified wavelet. TAFFYS provides a variety of wavelet families for wavelet analysis and the default sym8 wavelet used in TAFFYS can precisely reconstruct the abrupt breakpoint between segments. For a given decomposition level *N* (default as 6 in TAFFYS), the decomposition procedure iteratively generates two kinds of coefficients: detail coefficients (from the high-pass filter) and approximation coefficients (from the low-pass filter). The latters are further decomposed in next level with high- and low-pass filters, finally leading to a filter tree with one set of level *N* approximation coefficients and *N* sets of detail coefficients from level 1 to *N*.

The determination of threshold of detail coefficients: For each decomposition level, soft thresholding is adopted for retaining the indicative signal and eliminating the reflection of noise by setting the detail coefficients to 0. Based on a threshold determined by principle of Stein's Unbiased Risk Estimate (SURE), soft thresholding initially sets to zero the coefficients that have smaller values than the threshold, and then shrinks the nonzero coefficients toward 0.

The reconstruction of signal: According to the wavelet approximation coefficients from level N and the modified detail coefficients from all decomposition levels, the original signal is finally reconstructed. Generally, with a high value of composition level N, the noise will be significantly suppressed, leading to a small signal variance, as shown in S2 Fig.

### Detection method

To detect genomic aberrations from tumor SNP array data, our previous work proposed an efficient framework for tackling the issues of normal cell contamination and tumor aneuploidy that commonly occurs in tumor samples [10]. In this study, TAFFYS also includes this basic framework, but with a more sophisticated model for depicting complex LRR/BAF signal pattern in Affymetrix platform.

#### Hidden states definition

TAFFYS adopts total *S* = 20 hidden states for defining the possible aberrations in cancer genome, as illustrated in S1 Table. For the *i* ^*th*^ probe in the genome, we define the underlying tumor genotype *G* = (*m*_*i*,*t*_, *n*_*i*,*t*_) where *m*_*i*,*t*_ ∈ {0,…,*n*_*i*,*t*_} denotes the copy number of B allele and *n*_*i*,*t*_ is the total copy number. For instance, tumor genotype ‘ABB’ can be represented by *G* = (2,3). Similarly, *G* = (*m*_*i*,*n*_,*n*_*i*,*n*_) where *n*_*i*,*n*_ = 2 *m*_*i*,*n*_ ∈ {0, 2} corresponds to the normal genotype.

#### Emission probability function

Given the signal distributions discussed above, the overall emission probability can be calculated with joint probability density functions (*f*(*l*_*i*_|∼) and *f*(*b*_*i*_|∼)) for observed genotyping signals {*l*_*i*_, *b*_*i*_}, which can be written as follows:

$$f(l_{i},b_{i}|\theta ,s)=p_{f}f(l_{i})f(b_{i})+(1-p_{f})f(b_{i}|w,K,\sigma_{2}^{Bhom},\sigma^{Bhet},s)f(l_{i}|w,h,o,\sigma^{L},s)$$ (4)

here $\theta =\{w,h,o,\sigma^{L},K,\sigma_{2}^{Bhom},\sigma^{Bhet}\}$ denotes all the parameters in emission probability functions: *w* denotes the proportion of normal cells contaminated in the tumor sample, *o* is the correction factor for the shift of LRR baseline due to tumor aneuploidy, and *h* is the coefficient for local GC content *g*_*i*_. *σ* ^*L*^, $\sigma_{2}^{Bhom}$ and *σ* ^*Bhet*^ correspond to the respective STDs of LRR, homozygosity and heterozygosity BAF signals. *p*_*f*_ is the prior probability of signal fluctuation (default as 0.01), *f*(*l*_*i*_) and *f*(*b*_*i*_) correspond to the emission probability functions of fluctuated LRR and BAF signals, which are assumed to be uniformly distributed between [–5,5] and [0,1], respectively.

#### Transition matrix

A transition matrix is adopted in TAFFYS to measure the probability of aberration state transition, associated with initial matrix *A* ^*(0)*^ defined as follows:

$$A_{kl}^{\left(0\right)}=\left\{ \begin{array}{c} \frac{p_{t}}{S-1},k\neq l\\ 1-p_{t},k=l \end{array}\right.\;(k,l=1,\ldots ,S)$$ (5)

where $A_{kl}^{\left(0\right)}$ indicates the initial element of the transition matrix in *k* ^*th*^ row and *l* ^*th*^ column, *p*_*t*_ corresponds to the initial probability of transitions (default value is 10^−5^).

#### Parameters estimation

TAFFYS uses the expectation maximization (EM) algorithm for iteratively seeking the optimal parameter ***θ***. Generally, given the parameters estimate ***θ*** ^(*n*)^ at the *n* ^*th*^ iteration, the updated estimate ***θ*** ^(*n*+1)^ can be obtained by maximizing the expectation of log-likelihood of complete tumor SNP array data {***l*,*b***}:

$$\theta^{\left(n+1\right)}=\arg\;\max_{\theta }\;E_{l,b,\theta^{\left(n\right)}}[\log\;L(l,b,\theta )]$$ (6)

here *L*(***l*,*b*,*θ***) is the partial log-likelihood function for emission probability, which is given by:

$$L(l,b,\theta )=\sum_{i=1}^{N}\sum_{s=1}^{S}\;I_{i}(s)log\;[f(l_{i},b_{i}|\theta ,s)]$$ (7)

here *I*_*i*_(*s*) is the indicator function, which is equal to 1 when the *i* ^*th*^ SNP is in state *s*, otherwise 0. The expectation of the partial log-likelihood can be decomposed as follows:

$$E_{l,b,\theta^{\left(n\right)}}[\log\;L(l,b,\theta )]=\sum_{i=1}^{N}\sum_{s=1}^{S}(1-\gamma_{i,f}^{\left(n\right)}(s))\{log\;[f(l_{i}|w^{\left(n\right)},h^{\left(n\right)},o^{\left(n\right)},\sigma^{L(n)},s)]+$$

$$log[f(b_{i}|w^{\left(n\right)},K^{\left(n\right)},\sigma_{2}^{Bhom(n)},\sigma^{Bhet(n)},s)]\}+\gamma_{i,f}^{\left(n\right)}(s)\{\log\left[f(l_{i})\right]+log[f(b_{i})]\}$$ (8)

here $\gamma_{i,f}^{\left(n\right)}(s)$ corresponds to the conditional posterior probability of signal fluctuation, which is given by:

$$\gamma_{i,f}^{\left(n\right)}(s)=\gamma_{i}^{\left(n\right)}(s)\frac{p_{f}f(l_{i})f(b_{i})}{f(l_{i},b_{i}|\theta^{\left(n\right)},s)}$$ (9)

here $\gamma_{i}^{\left(n\right)}(s)$ corresponds to the posterior probability of the *i* ^*th*^ SNP belongs to state *s*, which is calculated by using the forward-backward algorithm.

To maximize the expectation of the partial log-likelihood, TAFFYS updates the parameters estimate ***θ*** ^(*n*+1)^, which consists of seven sub-procedures at each iteration.

For example, for parameter *K* ^(*n*+1)^, the update procedure is given by:

$$K^{\left(n+1\right)}=K^{\left(n\right)}-\frac{\frac{\partial E_{l,b,\theta^{\left(n\right)}}[\log\;L(l,b,\theta )]}{\partial K}}{\frac{\partial^{2}E_{l,b,\theta^{\left(n\right)}}[\log\;L(l,b,\theta )]}{{\partial K}^{2}}}$$ (10)

with

$$\frac{\partial E_{l,b,\theta^{\left(n\right)}}[\log\;L(l,b,\theta )]}{\partial K}=\sum_{i=1}^{N}\sum_{s=1}^{S}\left(1-\gamma_{i,f}^{\left(n\right)}(s))p_{i}(hom)(\frac{{\left(b_{i}-1\right)}^{2}(n_{i,t}(s)-2)}{{\left(\sigma_{2}^{Bhom(n+1)}\right)}^{2}e^{2K^{\left(n\right)}(n_{i,t}(s)-2)}}-(n_{i,t}(s)-2)\right)$$ (11)

$$\frac{\partial^{2}E_{l,b,\theta^{\left(n\right)}}[\log\;L(l,b,\theta )]}{{\partial K}^{2}}=\sum_{i=1}^{N}\sum_{s=1}^{S}\left(1-\gamma_{i,f}^{\left(n\right)}(s))p_{i}(hom)(-\frac{2{\left(b_{i}-1\right)}^{2}{\left(n_{i,t}(s)-2\right)}^{2}}{{\left(\sigma_{2}^{Bhom(n+1)}\right)}^{2}e^{2K^{\left(n\right)}(n_{i,t}(s)-2)}}\right)$$ (12)

where *p*_*i*_ *(hom)* denotes the prior probabilities of homozygous genotype at the *i* ^*th*^ probe, and it can be obtained by referring population frequency of B allele in PFB file (Details see S1 File).

The parameter estimation iteration will finally stop when the log-likelihood converges (the differential of log-likelihood between two adjacent iterations becomes less than 0.1%), and then the genomic aberrations and tumor genotypes are ascertained based on the posterior probabilities $\gamma_{i}^{\left(n\right)}(s)$ from the last iteration. Finally, based on the Eq (4), a goodness score for observed signal under the given state is calculated for each SNP, which can be used to reflect the discrepancy between observed and expected values. More details of methods are available in S1 File.

### Significance test

TAFFYS provides a permutation-based approach to evaluate the statistical significance of genome-wide aberrations in tumor samples, and summarized statistics is used to reflect copy number and frequency of each altered region in cancer genome. Generally, suppose there are multiple tumor samples available with sample size of *M* (*M*>1), and each sample contains *N* SNP probes across the whole genome. According the aberration types, we use the alteration scores $T_{i}^{amp}$, $T_{i}^{del}$ and $T_{i}^{LOH}$ to represent the test statistics at the *i* ^*th*^ probe for amplification, deletion and LOH, respectively. Specifically, the statistic $T_{i}^{amp}$ denotes the sum of amplification levels across all *M* samples in the set:

$$T_{i}^{amp}=\sum_{j=1}^{M}\;max(n_{i,j,t}-n_{i,j,n},0)$$ (13)

here, *n*_*i*,*j*,*t*_ and *n*_*i*,*j*,*n*_ correspond to the tumor and normal copy number at the *i* ^*th*^ probe for *j* ^*th*^ sample. Similar to statistic $T_{i}^{amp}$, test statistics $T_{i}^{del}$ and $T_{i}^{LOH}$ are also calculated.

To evaluate the statistically significant altered regions in cancer genome, TAFFYS adopts an exact test approach for statistics $T_{i}^{amp}$, $T_{i}^{del}$ and $T_{i}^{LOH}$. The null hypothesis is that aberrations randomly occur across the whole genome. The reference distribution of null hypothesis can be obtained by simulating all possible values of the test statistic under combinations of aberrations observed in cancer genome, which can be calculated by the convolution of histograms of statistics over all tumor samples. Specifically, for amplification, let $h_{j}^{amp}$ represents the histogram of statistic $T_{i}^{amp}$ for the *j* ^*th*^ tumor sample, and the exact null hypothesis distribution for all *M* samples is given by:

$$H^{amp}=h_{1}^{amp}⊗h_{2}^{amp}⊗\ldots ⊗h_{M}^{amp}$$ (14)

Furthermore, the probability of statistic $T_{i}^{amp}$ for underlying permutation test is given by

$$\mathrm{Pr}\left(T_{i}^{amp}\right)=\sum_{T:T>T_{i}^{amp}\;}Pr(H^{amp}(T))$$ (15)

here *Pr*(*H* ^*amp*^(*T*)) is the probability under the reference histogram *H* ^*amp*^ of a potential score *T* (also known as p-value), with larger score of *T* corresponding to notionally greater departure from null hypothesis. Similarly, the p-values for test statistics $T_{i}^{del}$ and $T_{i}^{LOH}$ can be calculated by this way. Furthermore, to produce relative conservative results with lower Type I error rate in multiple hypothesis testing, the p-values are further corrected by using FDR procedure in TAFFYS. The corrected probability, known as q-value, is finally used to ascertain statistically significant recurrent aberrations. More details of methods see S1 File.

### The TAFFYS software

TAFFYS provide a one-stop solution for a batch of Affymetrix SNP array sample analysis. It is fully automatic without any manual inspection or intervention. Genomic aberrations detected by TAFFYS and corresponding tumor genotypes are saved in result files, which also include summarized information regarding tumor SNP array data, such as normal contamination level, tumor average copy number and signal variances. To facilitate data analysis, TAFFYS provides visualization of identified genomic aberrations for each chromosome. Finally, statistical significance test is automatically performed to multiple tumor samples and the results are both visually and textually generated for further inspection. TAFFYS is implemented in standalone software package, and available from the associated website: http://bioinformatics.ustc.edu.cn/taffys/. The usage sees S2 File. Besides, this website also provides the LRR/BAF signal files pre-processed by PennCNV-affy and result files generated by TAFFYS. All these files can be freely downloaded by users.

## Results and Discussion

### Performance of wavelet-based de-noising

**Fig 3:**
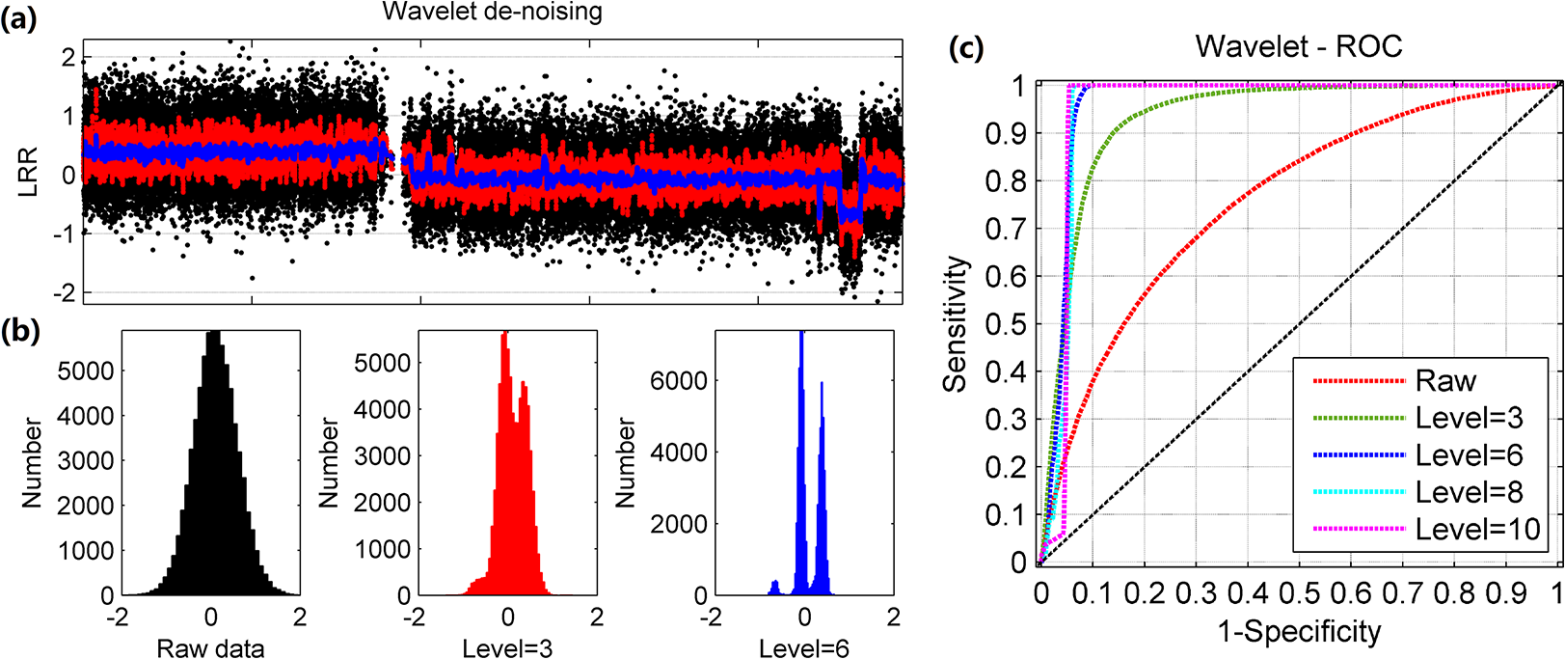
Assessment of wavelet de-noising on LRR signals. Assessment of performance of wavelet de-noising. (a) Comparison between raw LRR signals and recovered signals with decomposition level of 3 and 6. (b) Histograms of raw LRR signals and recovered signals with decomposition level of 3 and 6. (c)Assessment of quality improvement using ROC curves for recovered signals associated with decomposition level 3, 6, 8 and 10.

Signal de-noising is critical for subsequent genomic aberration detection, and the evaluation of de-noising approach depends on two aspects: noise suppression and recovery of original signals. Here, we first examine the quality of signal after performing wavelet-based signal de-noising. Fig 3A plots both raw and processed (decomposition levels of 3 and 6) LRR signals on chromosome 2 in cell-line sample H1395. With the aid of wavelet de-noising, the noise level in LRR signals is efficiently suppressed, leading to a consistent but more distinctive pattern of copy number alteration with breakpoint and centre of each signal band segment remaining unchanged. This result is also represented in the histograms of LRR signals (Fig 3B): owning to the enhanced SNR after de-noising, the three peaks representing different genomic aberrations on chromosome 2 become more discriminative as the decomposition level increases. Furthermore, we plot the receiver operating characteristic curve (ROC curve) to demonstrate the improvement of recovered signal associated with decomposition level (as shown in Fig 3C). It should be pointed out that ROC curves must be used with extreme caution unless one has a very large sample size [21]. In this study, more than 60,000 SNP probes on chromosome arm 2p and part of 2q (the end region with deletion is removed), which contain different LRR signal amplitudes, are selected for examination. Given any threshold, we calculate the true positive (TP, the number of the SNPs in chromosome 2p that above the threshold), false positive (FP, the SNPs in 2q that are above the threshold), true negative (TN, SNPs in 2q below the threshold) and false negative (FN, SNPs in 2p that below the threshold). By changing the threshold, a series of the sensitivities (SN) and specificities (SP) are obtained as follows: SN = TP/(TP+FN) and SP = TN/(FP+TN). The same procedure is repeated for raw and processed signals with different decomposition levels. The result of ROC curve indicates that at first discrimination of signals is apparently improved as the decomposition level gradually increases and best case occurs when the level is about 6. As the level keeps increasing, the performance decreases due to the over-de-nosing. Taking together, we come to the conclusion that the optimal wavelet de-noising decomposition level should be around 6 in practical application.

### Performance on lung cancer dilution series

To evaluate performance for aberration identification, we apply TAFFYS to a lung cancer cell-line dataset, which contains four tumor samples mixed with known proportions of matched normal cell-line. All these samples are hybridized to the Affymetrix GenomeWideSNP6.0 (GW6) genotyping array and raw CEL data files are available on GEO website with accession number [GEO: GSE29172]. The performance on this dataset reflects the recoverability of method against the normal cell contamination. Table 1 shows the detail parameters estimated by TAFFYS. Previous study on these samples revealed the cancer genome was highly altered and the tumor average copy number (ACN) was close to 3 [13], which clearly verifies the estimated results from TAFFYS. The consistency of tumor ACN estimation also suggests TAFFYS provides concordant genomic aberration identification results, and it is further confirmed by the genome-wide aberration profiles shown in S3 Fig. For BAF signals, both $\sigma_{2}^{Bhom}$ and *σ* ^*Bhet*^ are about 0.06 for all tested samples, which are significantly larger than the common STD of 0.03 for Illumina SNP arrays [10], suggesting a higher noise level perturbed in Affymetrix SNP arrays. Also the non-trivial increment coefficient *K* shows tumor copy number has considerable contributions on variance of Affymetrix SNP array signals.

**Table 1:** Parameters estimation on dilution series data.

Sample	ACN^#^	*o*	*h*	*σ* ^*Bhet*^	$\sigma_{2}^{Bhom}$	*K*
Tumor-100pc	2.80	-0.19	-0.01	0.06	0.06	-0.18
Tumor-70pc	2.82	-0.12	-0.02	0.07	0.05	-0.10
Tumor-50pc	2.78	-0.08	0.02	0.07	0.06	-0.08
Tumor-30pc	2.98	-0.06	0.03	0.05	0.06	-0.00

^#^:ACN = average copy number

**Fig 4:**
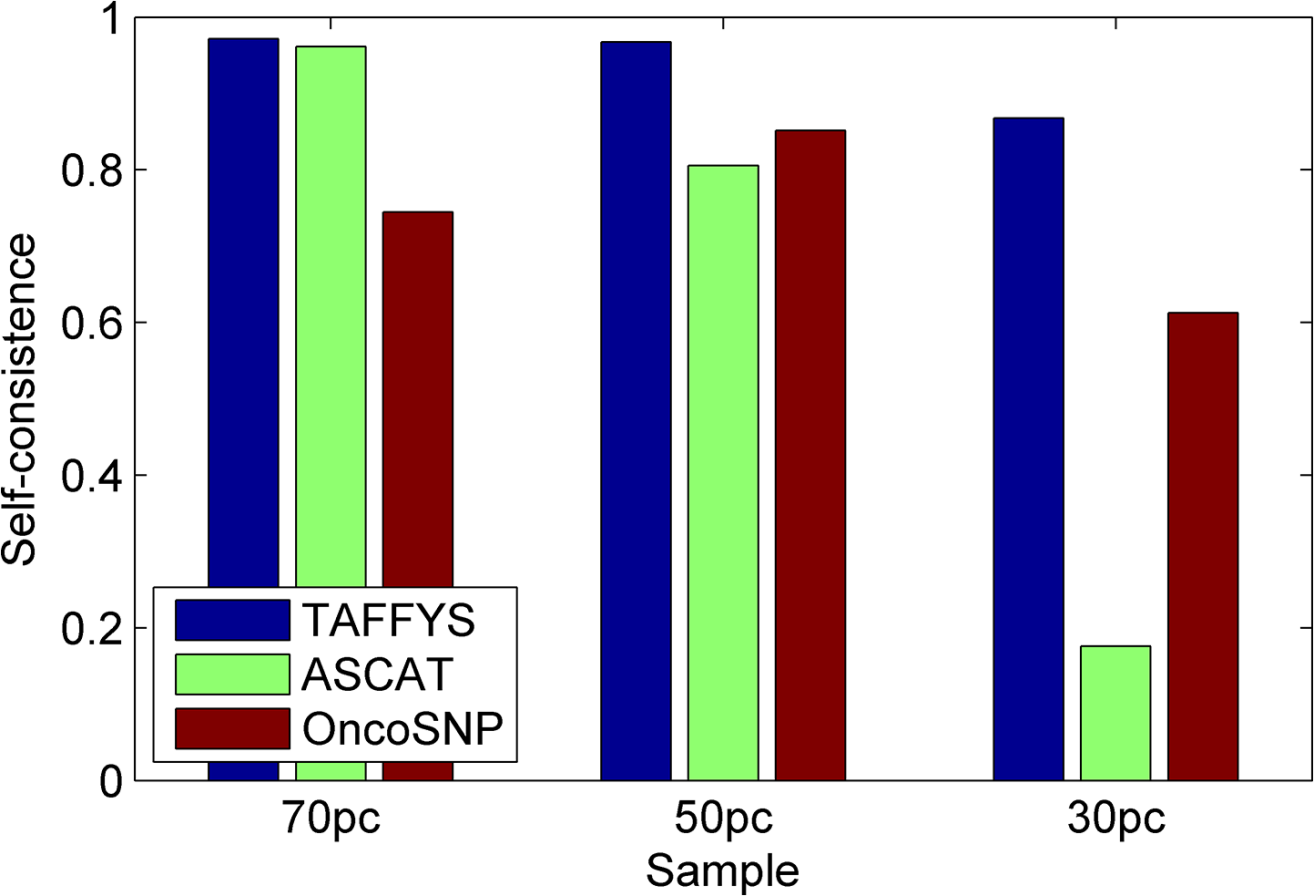
Assessment of different methods using dilution series data. Self-consistency for copy number identification by TAFFYS (blue), ASCAT (green) and OncoSNP (red).

**Fig 5:**
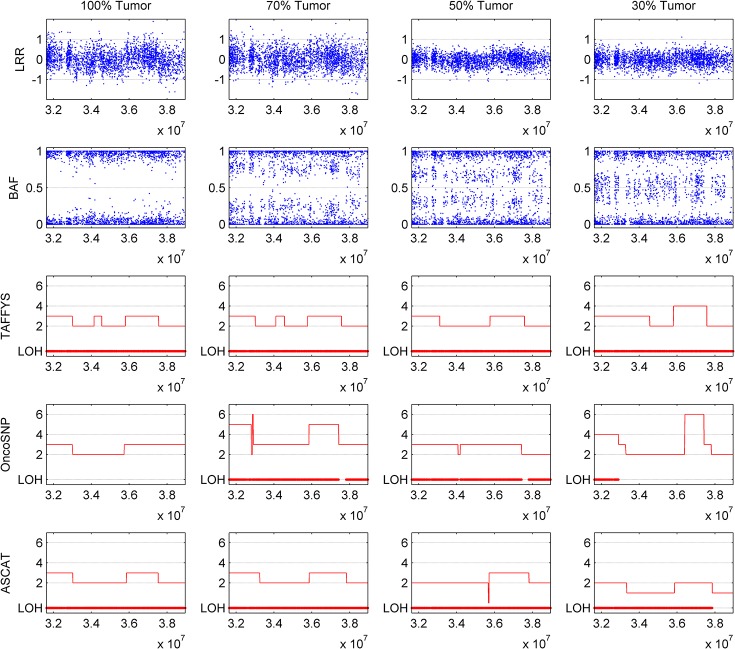
Comparison of results for lung tumor H1395 by different methods. Copy number and LOH results for chromosome 15 of four dilution samples with 100%, 70%, 50% and 30% cancer cells were generated by TAFFYS, ASCAT, and OncoSNP.

Next, we compare the performance of TAFFYS with two fully automatic state-of-the-art methods: OncoSNP and ASCAT. Similar with TAFFYS, both two methods parameterize the cancer cell content and signal baseline shift into integrated statistical models. On the other hand, instead of using EM algorithm, they prefer to find the optimal content value by grid search. Moreover, noise pattern in Affymetrix SNP array signals are not specially considered in their models. Paralleling our previous studies [9,10], here we calculate the self-consistencies for all three methods, which are defined as the proportion of SNPs in mixed sample, that have the same aberration types when comparing with pure tumor sample. The self-consistencies in Fig 4 show that TAFFYS outperforms other methods in all three mixture samples. Even when there are only 30% of cancer cells, TAFFYS still achieves more than 90% of self-consistency, suggesting its robustness to normal cell contamination. In comparison, OncoSNP has relatively low performance throughout all mixed samples, with self-consistency ranging from 60% to 80%. For ASCAT, it renders competitive results for lowly contaminated samples, but its performance sharply drops when the level of normal cell contamination continue to rise. Furthermore, we zoom in on a part of region to illustrate the detailed identification results of three methods. Fig 5 shows the aberration identification results of chromosome 15 on mixture samples. Although the noise significantly blurs the complex signal alterations in the aberration regions, TAFFYS accurately identifies short amplified regions from surrounding copy neutral LOH regions in all samples. In contrast, OncoSNP and ASCAT show less robustness and fail to detect the copy number alteration for highly contaminated samples. For example, ASCAT incorrectly predicts half of regions as hemizygous deletion for this sample.

Although TAFFYS does not provide direct indication for intra-tumor heterogeneity inference, alternatively, tumor subclones can be reflected by the goodness score of observed signal when given the aberration type determined in HMM model. This score will be noticeably lower if the distributions of genotyping signals do not fit to any pre-defined copy number levels, and thus the subclone regions can be identified. S4 Fig shows the result of chromosome arm 10q in tumor sample H1395 from TAFFYS. In order to give a clear illustration, we only plot the scores that are smaller than 0.05. The relative higher density of dots in end region of 10q indicates the possibility of tumor heterogeneity, and this result also corroborates the previous study reported the heterogeneity with the subclones of copy neutral state and deletion [13]. Correspondingly, TAFFYS provides a not perfect but reasonable interpretation for this region.

### Performance comparison on different platforms

**Fig 6:**
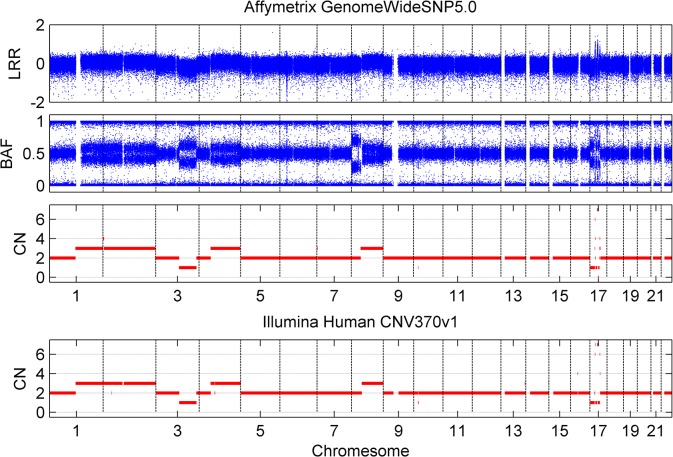
Comparison of results from TAFFYS and GPHMM. Comparison of genome-wide copy number profiles obtained from TAFFYS using Affymetrix GenomeWide5.0 SNP array and GPHMM copy number profile from Illumina HumanCNV370v1 SNP array analysis.

Affymetrix has released a series of SNP array platforms for human genome genotyping. Despite the differences in chip design, resolution and signal pre-process suites, they have been successfully applied into tumor samples analysis. Here, we employ real tumor samples to assess the performance on different Affymetrix SNP arrays. Firstly, we focus on the breast cancer sample 7204, which is both analyzed by Affymetrix GenomeWide5.0 (GW5) and Illumina HumanCNV370k [5]. This dataset is available on GEO website with the accession number [GEO: GSE16400]. The Affymetrix data is applied into TAFFYS to generate the aberration profile, compared with the result of Illumina SNP array data, which is processed by tQN [22] and GPHMM [10] for signal pre-processing and detection of genomic aberration. The genome-wide copy number aberrations are shown in Fig 6. The genomic aberration results of TAFFYS show excellent concordance with those of GPHMM. Furthermore, we compare the results of lung cancer cell-line sample H1395 on another two Affymetrix platforms: Affymetrix Mapping 500K (available on GEO website with accession number [GEO: GSE17247]) and Affymetrix GW6.0. The results in S5 Fig demonstrate that TAFFYS still yields very agreeable results on both Affymetrix platforms. Taken together, these results suggest TAFFYS provides reliable detection of genomic aberration on different Affymetrix SNP arrays.

### Discovery of significantly altered regions on cancer genome

**Fig 7:**
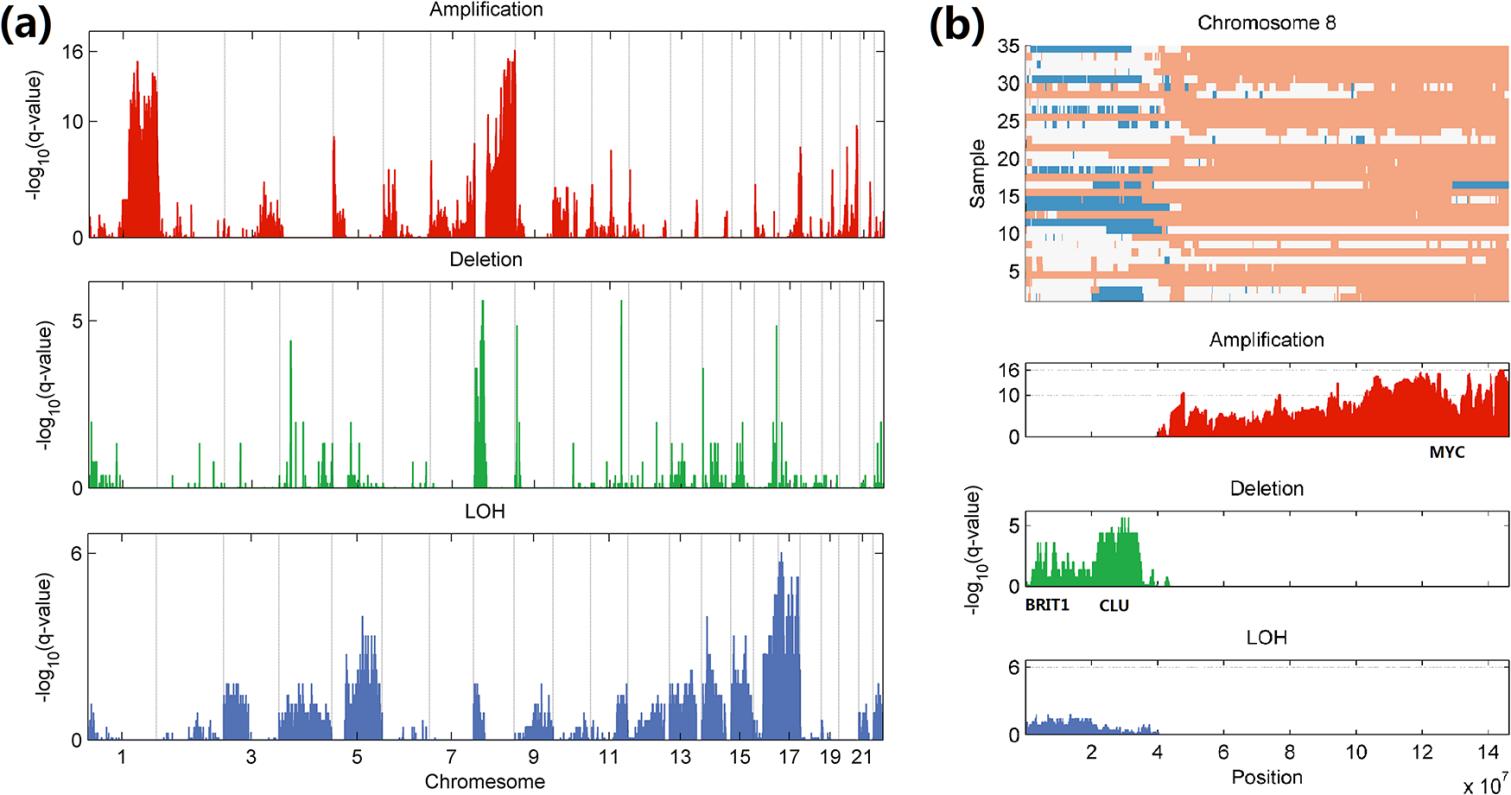
Genome-wide analysis of statistically significant aberrations on a collection of breast tumors. (a) Statistical significance for amplification (red), deletion (green) and LOH (blue) are evaluated by q-value. (b) Detailed visualization of aberration profiles of amplification(red)/deletion(blue) and statistical significance on chromosome 8.

Finally, we download 44 breast cancer samples from the GEO website (accession number [GEO: GSE26232]) and evaluate the statistical significance of genomic aberrations by using TAFFYS. Due to the inevitable factors in experiment process and sample quality, some SNP array samples are likely to present very poor signal quality, and this becomes particular common during the analysis of a batch of samples. Compared with semi-automatic methods, one feature of TAFFYS is its quantitative model with fully automatic estimation of related parameters, which can assist the user’s effort in manual inspection for the quality control. S2 Table shows the detail information of 9 out of 44 samples, which are eliminated by TAFFYS from further analysis. For most of them, the higher noise and GC related bias are main reasons leading their low signal qualities. Next, the statistical significance of aberration region is assessed by using the rest of 35 breast cancer samples, and the results are shown in Fig 7A. High significances are obtained in regions of chromosome 1, 5, 8, 17 and etc. Moreover, Fig 7B shows the detailed amplification/deletion profile of chromosome 8 and visualized q-values generated from significance test, and it clearly demonstrate significantly amplified and deleted regions on 8q and 8p, respectively. For example, as one of the most significantly amplified region (average q-value <10^−8^), 8q24 harbors an important oncogene MYC, which has been proven to play a critical role in the carcinogenesis of breast cancer. Also from the results of significant deletions on 8p, we identify many well-known cancer related genes including BRIT1 (average q-value <10^−5^) and CLU (average q-value <10^−5^) [23]. On the other hand, the results of significance test suggest there is no recurrent LOH region on chromosome 8, indicating both two alleles ‘A’ and ‘B’ alters without growth advantage over each other in these breast cancer tumors.

## Discussion

One of apparent differences between Affymetrix and Illumina SNP array is the raw genotyping signals pre-processing. For Affymetrix raw data, namely.CEL file, should be pre-processed to normalize and extract the LRR and BAF signals. So far, there are several tools available for doing this, including commercial and non-commercial ones [15,24,25]. Although they are generally robust to the chip effects, raw signal noise, there are large differences in operating environment, output format, and most importantly, the quality of normalized signals. In previous study, a comprehensive comparison was conducted for evaluating the variant of current non-commercial tools, and results suggested that PennCNV-affy [6] had relatively small bias and variability [26]. Therefore, PennCNV-affy is chose as the default pre-processing tool for TAFFYS. Besides, in this study we compare our method with ASCAT and OncoSNP for copy number identification. Another method TAPS, which is also proposed for Affymetrix SNP array data analysis [13], is not involved in performance comparison as its results are associated with parameters assigned by users. Instead of using a fully automatic statistical model, TAPS first generate a signal distribution scatter plot, with LRR and BAF as horizontal and vertical axis respectively. Manual inspection is then needed to determine difference of signal between copy numbers, which will be eventually used to calculate cancer cell content and detect the copy number alteration. It should also be noted that this strategy has provided an efficient solution for reducing the statistical uncertainty of models and identifying tumor heterogeneity regions.

TAFFYS produces goodness scores for describing how well identified aberrations fit observed signals. In this study, we find that by inspecting the goodness scores, TAFFYS can be used to identify the tumor heterogeneity regions. Currently, several approaches are designed to provide such heterogeneity-related score for each interrogated probe [7,17,26], and it represents a simple and efficient strategy for inferring the intra-heterogeneity in SNP array. However, to the best of our knowledge, none of them can explicitly de-convolute the essential information of intra-tumor heterogeneity, such as the total number of subclone and aberration specified for each subclone. We advocate the development of more sophisticated models will fill this gap.

By combining the information of dbSNP id, position and chromosome provided by raw CEL file, user can easily match TAFFYS copy number profile with the interested genes by querying online database, e.g. UCSC Genome Browser. However, due the limited resolution of SNP array data, parts of genome, such as promoter regions, are not covered in Affymetrix SNP array. Moreover, we can only use copy number information from SNP to estimate the copy number of gene that override this SNP. Currently, NGS technology gains its popularity in recent researches on cancer genome. The high resolution of NGS, leveraged with enormous demands in storage and computation presents a challenging task for the genomic studies[26]. Despite of the disadvantages of SNP arrays, they are still irreplaceable at present especially considering the low costs, wide availability in publicly database. Also for some NGS data processing methods, Affymetrix SNP array serves as a golden standard for validating the performance [27,28]. Due to the differences in raw data pre-process and signal statistical distribution [29], TAFFYS is not directly applicable for NGS data analysis at present. However, considering the similarity of measurements between SNP array and NGS [28,29], some methodologies in the TAFFYS can be easily porting into NGS data analysis tools. For example, the strategy for suppressing the noise may also provide an efficient framework for processing the NGS signals. We are now actively extending our method into a more general tool with support of NGS data analysis

## Conclusions

We describe a bioinformatic tool, named TAFFYS, for automatic identification of copy number alteration and allelic imbalance using Affymetrix tumor SNP array data. The applications on different tumor dataset show that TAFFYS can provide accurate interpretation for the genomic aberrations even when the tumour sample is severely contaminated by normal cells. Besides, statistical significance test on a collection of breast cancer samples provides a comprehensive characterization of recurrent aberrations on the cancer genome. In conclusion, we believe that TAFFYS will be an efficient tool for tumor Affyemtrix SNP array analysis, and assist the research on genomic aberration identification and recurrent aberration assessment.

## Supporting Information

S1 File Detailed description of statistical methods in TAFFYS. (PDF)

S2 File Usage of TAFFYS. (PDF)

S1 Fig The entire pipeline of TAFFYS. (PDF)

S2 Fig Performance evaluation of wavelet de-noising on LRR signals. (a) Results of processed LRR signals at different wavelet decomposition levels, including 2, 3, 5 and 6. (b) Illustration of corresponding LRR variances at different decomposition levels.
(PDF)

S3 Fig Assessment of genomic aberration identification for TAFFYS using dilution series data. Genome-wide amplification (red) /deletion (blue) profiles for dilution samples with cancer cell content ranging from 30% to 100%.
(PDF)

S4 Fig Aberration identification of TAFFYS using lung cancer H1395. The results of genome-wide aberration identification on chromosome 10 using lung cancer H1395. For the BAF panel, LOH region is marked with blue, while non-LOH region with gray. For LRR panel, amplification is colored with red, and deletion with green. Black dots denote the signals after performing de-noising. For copy number (CN) panel, red line correspond to the copy number, and blue dots denote the goodness scores, which are only plotted when they are smaller than 0.05.
(PDF)

S5 Fig Comparison of genomic aberration identification of TAFFYS between GenomeWideSNP6.0 and Mapping500k. The results of genome-wide aberration identification using lung cancer H1395, which are analyzed by TAFFYS using Affymetrix GenomeWideSNP6.0 (bottom) and Affymetrix Mapping 500k (top).
(PDF)

S1 Table Detailed information of hidden states in TAFFYS. (PDF)

S2 Table Detailed information of eliminated samples. (PDF)
