# Supplementary material for: TAFFYS: An Integrated Tool for Comprehensive Analysis of Genomic Aberrations in Tumor Samples
Source: PLoS One. 2015 Jun 25;10(6):e0129835. doi: 10.1371/journal.pone.0129835 (PMC4482394; doi:10.1371/journal.pone.0129835)
Supplement: S1 File — (PDF) [file pone.0129835.s001.pdf]

TAFFYS: an integrated tool for comprehensive analysis of genomic aberrations in tumor samples using Affymetrix SNP arrays

(Supplemental material)

● Software introduction

TAFFYS provides an integrated analysis for genomic aberrations and Figure S1 shows the entire pipeline, which includes signal pre-processing, aberration detection and significance test. TAFFYS takes Affymetrix raw CEL file as input and is fully automatic without any manual monitoring or intervention. Genomic aberrations detected by TAFFYS and corresponding tumor genotypes are saved in result files, which also include summarized information regarding tumor SNP array data, such as normal contamination level, tumor average copy number (ACN) and signal variances. To facilitate data analysis, TAFFYS provides visualization of identified genomic aberrations for each chromosome. Finally, statistical significance test is automatically performed to multiple tumor samples and the results are both visually and textually generated for further inspection. TAFFYS is implemented in standalone Matlab/C package, and available from the associated website: http://bioinformatics.ustc.edu.cn/taffys/.

● Method

1. Overview

TAFFYS offers an integrated solution for Affymetrix tumor SNP array data analysis. First, Affymetrix CEL file is pre-processed to extract raw genotyping signals. The PennCNV-affy [1] built-in module transforms the normalized signals into two measurements: Log R Ratio (LRR) and B Allele Frequency (BAF) [2, 3], which denote the relative total copy number and the fraction of B allele, respectively. A wavelet de-noising procedure [4] is then applied to suppressing noise of LRR signals. Based on the statistical distributions of the LRR and BAF signals, TAFFYS adopts a

hidden Markov model (HMM) and expectation maximization (EM) algorithm for inferring genomic aberration and tumor genotypes. Critical issues including signal variances, normal cell contamination, LRR baseline shift and GC content bias are quantitatively modeled and estimated. Finally, for multiple tumor samples TAFFYS provides a permutation-based approach to evaluate the statistical significance of aberrations in cancer genome.

2. Statistical distributions of Affymetrix genotyping signals

2.1. BAF signals

As the first step, we investigated BAF signals for Affymetrix platform, and Figure 2a illustrates the distributions of BAF signals with respect to different copy numbers for a lung cancer cell-line sample H1395. In contrast to the consistent signal variance in Illumina platform [5], the variance of BAF signals associated with homozygous tumor genotypes, e.g. 'B' and ''AAAA', dramatically rises when tumor copy number decreases. Further examination (Figure 2b) shows this relationship can be approximately modeled by a log-linear function:

$$log\left(\sigma_{n_t}^{Bhom}\right) - log\left(\sigma_2^{Bhom}\right) = K(n_t - 2) \quad (1)$$

here σ_2^{Bhom} is the standard deviation (STD) of BAF signals for diploid tumor genotypes, and $\sigma_{n_t}^{Bhom}$ is the STD of BAF signal associated with copy number $\mathrm{n_t}$. The slope of the fitted line, *K*, represents an increment coefficient against tumor copy number $\mathrm{n_t}$. This equation can be further written as:

$$\sigma_{n_t}^{Bhom} = \sigma_2^{Bhom} e^{K(n_t-2)} \quad (2)$$

Equation (2) will be used in the hidden Markov model for detection of genomic aberrations (see Section 3.2). In addition, to reduce computational complexity, BAF signals are upward mirrored along the 0.5 axis in TAFFYS.

2.2. LRR signals

Next, the statistical distributions of LRR signals for cell-line sample H1395 were investigated and shown in Figure 2c. The variance of Affymetrix LRR signals is consistent (~0.17) for different copy numbers and is about 3 times larger than that of Illumina platform (~0.04) [5]. Such high noise level creates an obstacle to precisely detecting genomic aberrations. To address this issue, a de-noising pre-processing procedure is adopted in TAFFYS to increase the SNR of LRR signals (see next

section). At the same time, Figure 2d shows the mean of LRR signals for Affymetrix platform does not fit to a previously proposed empirical formula for Illumina platform [3], and therefore we propose a modified formula for Affymetrix platform by adding a contraction coefficient β:

$$\mathrm{mean}(l) = \beta * log_{10}(\frac{n_t}{2}) \quad (3)$$

here l represents LRR signals associated with copy number $\mathrm{n_t}$. As illustrated in Figure 2d, by selecting an appropriate β Equation (3) can accurately delineate the statistical behavior of LRR signals when copy number is altered.

2.3. Wavelet-based signal de-noising

As discussed above, the issue of low SNR of Affymetrix LRR signals hampers interpretation of tumor SNP array data and therefore should be addressed before further data modeling and analysis. Generally, LRR signals represent a mixture of block signals with additive white Gaussian noise, featured by distinct aberration regions and sharp changes of LRR signals at the breakpoint of two adjacent regions. To suppress the noise and meanwhile recovery the original LRR signals, TAFFYS adopts a de-noising pre-processing procedure based on wavelet [4], which mainly contains three steps:

1) The decomposition of wavelet signal

Firstly, the wavelet transform decomposes each level of signals with two complementary high- and low-pass filters determined by the selected wavelet. TAFFYS provides a variety of wavelet families for wavelet analysis and the default sym8 wavelet used in TAFFYS can precisely reconstruct the abrupt breakpoint between two segments. For a given decomposition level *N*, the decomposition procedure iteratively generates two kinds of coefficients: detail coefficients (from the high-pass filter) and approximation coefficients (from the low-pass filter). The latters are further decomposed in next level with high- and low-pass filters, finally leading to a filter tree with one set of level *N* approximation coefficients and *N* sets of detail coefficients from level 1 to *N*.

2) The determination of threshold of detail coefficients

For each decomposition level, soft thresholding is adopted for retaining the indicative signal and eliminating the reflection of noise by setting the detail coefficients to 0. Based on a threshold determined by principle of Stein's Unbiased Risk Estimate

(SURE), soft thresholding initially sets the coefficients that have smaller values than the threshold to zero, and then shrinks the nonzero coefficients toward 0.

3) The reconstruction of signal

According to the wavelet approximation coefficients from level N and the modified detail coefficients from all decomposition levels, the original signal is finally reconstructed.

It should be pointed out that the higher decomposition level usually results in lower noise level in processed signals while original signals may be deteriorated at the same time. In order to reduce the noise level in LRR signals and retain genotyping signals, we performed a systematic examination of signal variance against the decomposition level (Figure S2). When the decomposition level is equal to 6, most of the noise in LRR signals is removed. Therefore it is used as the default value in the implementation of TAFFYS.

3. Detection method

To detect genomic aberrations from tumor SNP array data, TAFFYS includes a hidden Markov model that can not only quantitatively model complex LRR and BAF signals for Affymetrix platform but also tackle the issues of normal cell contamination and tumor aneuploidy that commonly occur in tumor samples.

3.1. Hidden states definition

TAFFYS adopts total $S = 20$ hidden states for defining the possible aberrations in cancer genome, as illustrated in Table S1. For the ith probe in the genome, we define the underlying tumor genotype $G = (m_{i,t}, n_{i,t})$ where $m_{i,t} \in \{0, \dots, n_{i,t}\}$ denotes the copy number of B allele and $n_{i,t}$ is the total copy number. For instance, tumor genotype 'ABB' can be represented by $G = (2,3)$. Similarly, $G = (m_{i,n}, n_{i,n})$ where $n_{i,n} = 2$ $m_{i,n} \in \{0, 2\}$ corresponds to the normal genotype.

3.2. Emission probability function

To describe mean value of LRR signals with the existence of tumor aneuploidy and normal cell contamination, Equation(3) can be further extended as follows:

$$\mathrm{mean}(l_i) = \beta * log_{10}\left(\frac{y_i}{2}\right) + o + hg_i \qquad (4)$$

with

$$y_i = n_{i,n} * w + n_{i,t} * (1 - w) \tag{5}$$

here l_i represents the observed LRR signal at the ith probe and y_i represents average copy number with w being the proportion of normal cells contaminated in the tumor sample. o is the correction factor for the shift of LRR baseline due to tumor aneuploidy, and h is the coefficient for local GC content g_i that may also affect LRR signals. Furthermore, by assuming LRR signals are normally distributed with variance of σ^L, the emission probability of l_i for hidden state $s \in (1, \ldots 20)$ can be written as :

$$f(l_i|w, h, o, \sigma^L, s) = \frac{1}{\sigma^L} \phi(\frac{l_i - (\beta * log_{10}\left(\frac{n_{i,n}(1-w) + n_{i,t}(s)w}{2}\right) + o + hg_i)}{\sigma^L}) \tag{6}$$

here $\phi(\sim)$ is the probability density function of standard normal distribution.

For BAF signals, let σ_2^{Bhom} and σ^{Bhet} be the STD of BAF signals for diploid homozygous ($G = (m_{i,t}, n_{i,t})$ where $n_{i,n} = 2$ $m_{i,n} \in \{0, 2\}$) and heterozygous genotypes ($G = (m_{i,t}, n_{i,t})$ where $m_{i,n} \in \{1, \ldots, n_{i,n} - 1\}$) in tumor sample, respectively. For the observed BAF signal b_i at the ith probe for hidden state $s \in (1, \ldots 20)$, the emission probability can be derived from previously proposed formula [3] and equation(2), which can be written as follows :

$$f\left(b_i \middle| w, K, \sigma_2^{Bhom}, \sigma^{Bhet}, s\right) =$$

$$p_i(hom) \frac{1}{\sigma_2^{Bhom} e^{K\left(n_{i,t}(s)-2\right)}} \phi\left(\frac{b_i - (1)}{\sigma_2^{Bhom} e^{K\left(n_{i,t}(s)-2\right)}}\right) + p_i(het) \frac{1}{\sigma^{Bhet}} \phi(\frac{b_i - (z_i(s)/y_i(s))}{\sigma^{Bhet}}) \tag{7}$$

with

$$z_i = m_{i,n} * w + m_{i,t} * (1 - w) \tag{8}$$

here $p_i(hom)$ and $p_i(het)$ are the prior probabilities of homozygous and heterozygous genotype at the ith probe, which can be obtained from the population B allele frequencies in the PFB file (Figure S1). z_i is average copy number of B alleles. Taken together, the overall emission probability of observed genotyping signals $\{l_i, b_i\}$ is calculated as follows:

$$f(l_i, b_i|\boldsymbol{\theta}, s) =$$

$$p_f f(l_i) f(b_i) + \left(1 - p_f\right) f\left(b_i \middle| w, K, \sigma_2^{Bhom}, \sigma^{Bhet}, s\right) f(l_i|w, h, o, \sigma^L, s) \tag{9}$$

here $\boldsymbol{\theta} = \{w, h, o, \sigma^L, K, \sigma_2^{Bhom}, \sigma^{Bhet}\}$ denotes all the parameters in emission probability functions. p_f is the prior probability of fluctuation signal (default is 0.01), and $f(l_i)$ and $f(b_i)$ correspond to the emission probability functions of fluctuated

LRR and signals, which are assumed to be uniformly distributed between [-5, 5] and [0,1], respectively.

3.3. Transition matrix

A transition matrix is adopted in TAFFYS to measure the probability of aberration state transition, associated with initial matrix A^0 defined as follows:

$$A_{kl}^{(0)} = \begin{cases} \frac{p_t}{S-1}, k \neq l \\ 1 - p_t, k = l \end{cases} \quad (k, l = 1, \dots, S) \tag{10}$$

where $A_{kl}^{(0)}$ indicates the initial element of the transition matrix in k^{th} row and l^{th} column, p_t corresponds to the initial probability of transitions (default value is 10^{-5}). S is the number of all hidden state.

3.4. Parameters estimation

TAFFYS uses the expectation maximization (EM) algorithm for iteratively seeking the optimal parameter $\boldsymbol{\theta}$. Generally, given the parameters estimate $\boldsymbol{\theta}^{(n)}$ at the nth iteration, the updated estimate $\boldsymbol{\theta}^{(n+1)}$ can be obtained by maximizing the expectation of log-likelihood of complete tumor SNP array data $\{\boldsymbol{l}, \boldsymbol{b}\}$:

$$\boldsymbol{\theta}^{(n+1)} = arg\, max_{\boldsymbol{\theta}}\, \mathbb{E}_{\boldsymbol{l},\boldsymbol{b},\boldsymbol{\theta}^{(n)}}[log\, L(\boldsymbol{l}, \boldsymbol{b}, \boldsymbol{\theta})] \tag{11}$$

here $L(\boldsymbol{l}, \boldsymbol{b}, \boldsymbol{\theta})$ is the partial log-likelihood function for emission probability, which is given by:

$$L(\boldsymbol{l}, \boldsymbol{b}, \boldsymbol{\theta}) = \sum_{i=1}^{N} \sum_{s=1}^{S} I_i(s) log\, [f(l_i, b_i | \boldsymbol{\theta}, s)] \tag{12}$$

here $I_i(s)$ is the indicator function, which is equal to 1 when the ith SNP is in state s, otherwise 0. The expectation of the partial log-likelihood can be decomposed as follows:

$$\mathbb{E}_{\boldsymbol{l},\boldsymbol{b},\theta^{(n)}}[log\, L(\boldsymbol{l}, \boldsymbol{b}, \boldsymbol{\theta})]$$

$$= \sum_{i=1}^{N} \sum_{s=1}^{S} \left(1 - \gamma_{i,f}^{(n)}(s)\right) \{log\, \left[f\left(l_i \middle| w^{(n)}, h^{(n)}, o^{(n)}, \sigma^{L(n)}, s\right)\right] +$$

$$log[f\left(b_i \middle| w^{(n)}, K^{(n)}, \sigma_2^{Bhom(n)}, \sigma^{Bhet(n)}, s\right)]\} + \gamma_{i,f}^{(n)}(s)\{log[f(l_i)] + log[f(b_i)]\} \tag{13}$$

here $\gamma_{i,f}^{(n)}(s)$ corresponds to the conditional posterior probability of signal fluctuation, which is given by:

$$\gamma_{i,f}^{(n)}(s) = \gamma_i^{(n)}(s)\frac{p_f f(l_i) f(b_i)}{f(l_i, b_i|\boldsymbol{\theta}^{(n)}, s)} \quad (14)$$

here $\gamma_i^{(n)}(s)$ corresponds to the posterior probability of the ith SNP belongs to state s, which is calculated by using the forward-backward algorithm.

To maximize the expectation of the partial log-likelihood, TAFFYS updates the parameters estimate $\boldsymbol{\theta}^{(n+1)}$, which consists of seven sub-procedures at each iteration:

The EM update for the baseline shift $o^{(n+1)}$ is given by:

$$o^{(n+1)} = \frac{\sum_{i=1}^{N}\sum_{s=1}^{S}\left(1-\gamma_{i,f}^{(n)}(s)\right)\left(l_i - \left(\beta * log_{10}\left(\frac{y_i^{(n)}(s)}{2}\right) + h^{(n)} g_i\right)\right)}{\sum_{i=1}^{N}\sum_{s=1}^{S}\left(1-\gamma_{i,f}^{(n)}(s)\right)} \quad (15)$$

Where $y_i^{(n)}(s)$ corresponds to the weighted copy number level at the nth iteration.

The EM update for the GC content coefficient $h^{(n+1)}$ is given by:

$$h^{(n+1)} = \frac{\sum_{i=1}^{N}\sum_{s=1}^{S}\left(1-\gamma_{i,f}^{(n)}(s)\right) g_i \left(l_i - \left(\beta * log_{10}\left(\frac{y_i^{(n)}(s)}{2}\right) + o^{(n+1)}\right)\right)}{\sum_{i=1}^{N}\sum_{s=1}^{S}\left(1-\gamma_{i,f}^{(n)}(s)\right) {g_i}^2} \quad (16)$$

The EM update for the STD of LRR signal $\sigma^{L(n+1)}$ is given by:

$$\sigma^{L(n+1)} = \left(\frac{\sum_{i=1}^{N}\sum_{s=1}^{S}\left(1-\gamma_{i,f}^{(n)}(s)\right)\left(l_i - \left(\beta * log_{10}\left(\frac{y_i^{(n)}(s)}{2}\right) + o^{(n+1)} + h^{(n+1)} g_i\right)\right)^2}{\sum_{i=1}^{N}\sum_{s=1}^{S}\left(1-\gamma_{i,f}^{(n)}(s)\right)}\right)^{\frac{1}{2}} \quad (17)$$

The EM update for the STD of homozygous BAF signal ${\sigma_2}^{Bhom(n+1)}$ is given by:

$${\sigma_2}^{Bhom(n+1)} = \left(\frac{\sum_{i=1}^{N}\sum_{s=1}^{S}\left(1-\gamma_{i,f}^{(n)}(s)\right) p_i(hom)(b_i-1)^2}{\sum_{i=1}^{N}\sum_{s=1}^{S}\left(1-\gamma_{i,f}^{(n)}(s)\right) p_i(hom) e^{2K^{(n)}(n_{i,t}(s)-2)}}\right)^{\frac{1}{2}} \quad (18)$$

Similarly, the EM update for the STD of heterozygous BAF signal $\sigma^{Bhet(n+1)}$ is given by:

$$\sigma^{Bhet(n+1)} = \left(\frac{\sum_{i=1}^{N}\sum_{s=1}^{S}\left(1-\gamma_{i,f}^{(n)}(s)\right) p_i(het)\left(b_i - ({z_i}^{(n+1)}(s)/{y_i}^{(n+1)}(s))\right)^2}{\sum_{i=1}^{N}\sum_{s=1}^{S}\left(1-\gamma_{i,f}^{(n)}(s)\right) p_i(het)}\right)^{\frac{1}{2}} \quad (19)$$

Furthermore, considering the practice difficulty in deducing the close-form functions, TAFFYS adopts the Newton-Raphson method for updating the parameters $w^{(n+1)}$ and $K^{(n+1)}$. For example, for parameter $K^{(n+1)}$, the update procedure is given by:

$$K^{(n+1)} = K^{(n)} - \frac{\frac{\partial \mathbb{E}_{\boldsymbol{l},\boldsymbol{b},\boldsymbol{\theta}^{(n)}}[log\ L(\boldsymbol{l},\boldsymbol{b},\boldsymbol{\theta})]}{\partial k}}{\frac{\partial^2 \mathbb{E}_{\boldsymbol{l},\boldsymbol{b},\boldsymbol{\theta}^{(n)}}[log\ L(\boldsymbol{l},\boldsymbol{b},\boldsymbol{\theta})]}{\partial k^2}} \tag{20}$$

with

$$\frac{\partial \mathbb{E}_{\boldsymbol{l},\boldsymbol{b},\boldsymbol{\theta}^{(n)}}[log\ L(\boldsymbol{l},\boldsymbol{b},\boldsymbol{\theta})]}{\partial k} = \sum_{i=1}^{N}\sum_{s=1}^{S}\left(1-\gamma_{i,f}^{(n)}(s)\right)p_i(hom)\left(\frac{(b_i-1)^2(n_{i,t}(s)-2)}{\left(\sigma_2^{Bhom\ (n+1)}\right)^2 e^{2K^{(n+1)}(n_{i,t}(s)-2)}} - (n_{i,t}(s)-2)\right) \tag{21}$$

$$\frac{\partial^2 \mathbb{E}_{\boldsymbol{l},\boldsymbol{b},\boldsymbol{\theta}^{(n)}}[log\ L(\boldsymbol{l},\boldsymbol{b},\boldsymbol{\theta})]}{\partial k^2} = \sum_{i=1}^{N}\sum_{s=1}^{S}\left(1-\gamma_{i,f}^{(n)}(s)\right)p_i(hom)\left(-\frac{2(b_i-1)^2(n_{i,t}(s)-2)^2}{\left(\sigma_2^{Bhom\ (n+1)}\right)^2 e^{2K^{(n+1)}(n_{i,t}(s)-2)}}\right) \tag{22}$$

The parameter estimation iteration will finally stop when the log-likelihood converge (the differential of log-likelihood between two adjacent iterations becomes less than 0.1%), and then the genomic aberrations and tumor genotypes across the genome are ascertained based on the posterior probabilities $\gamma_i^{(n)}(s)$ from the last iteration. In order to find the optimal parameters for state transition, TAFFYS adopts the standard Baum Welch algorithm to update the elements of transition matrix. Finally, based on the Equation (9), a goodness score for observed signal under the given state is calculated for each SNP, which can be used to reflect the discrepancy between observed and the expected values.

4. Significance test

GISTIC [6] introduces an effective framework for assessing the recurrent aberrations by using permutation test. However, the raw genotyping signals which GISTC is directly imposed on, are very susceptible to the noise, normal cell contamination and other issues, and may finally lead to an inaccurate identification of significance aberration region. To facilitate systematic studies of recurrent and functionally important driver aberrations with multiple tumor samples, TAFFYS provides a permutation-based approach to evaluate the statistical significance of genome-wide aberrations in tumor samples, which uses summarized statistics to reflect copy number and frequency of each altered region in cancer genome.

Generally, suppose there are multiple tumor samples available with sample size of M (M>1), and each sample contains N SNP probes across the whole genome. According the aberration types, we use the alteration score T_i^{amp}, T_i^{del} and T_i^{LOH} to represent the test statistic at the ith probe for amplification, deletion and LOH, respectively. Specifically, the statistic T_i^{amp} denotes the sum of amplification levels across all M samples in the set:

$$T_i^{amp} = \sum_{j=1}^{M} max(n_{i,j,t} - n_{i,j,n}, 0) \qquad (23)$$

here, $n_{i,j,t}$ and $n_{i,j,n}$ correspond to the tumor and normal copy number at the ith probe for j^{th} sample. Similar to statistic T_i^{amp}, test statistic T_i^{del} and T_i^{LOH} are also calculated using sum representation, which are given by:

$$T_i^{del} = \sum_{j=1}^{M} max\left(n_{i,j,n} - n_{i,j,t}, 0\right) \qquad (24)$$

and

$$T_i^{LOH} = \sum_{j=1}^{M} L_{i,j,t} \qquad (25)$$

here $L_{i,j,t}$ denotes the tumor LOH state, which is equal to 1 when tumor aberration belongs to the LOH state, otherwise 0.

To evaluate the statistically significant altered regions in cancer genome, TAFFYS adopts an exact test approach for statistics T_i^{amp}, T_i^{del} and T_i^{LOH}. The null hypothesis is that all aberrations are passenger aberrations and randomly occur across the whole genome. The reference distribution of null hypothesis can be obtained by simulating all possible values of the test statistic under combinations of aberrations observed in cancer genome, which can be calculated by the convolution of histograms of statistics over all tumor samples. Specifically, for amplification, let h_j^{amp} represents the histogram of statistic T_i^{amp} for the jth tumor sample, and the exact null hypothesis distribution for all M samples is given by:

$$H^{amp} = h_1^{amp} \otimes h_2^{amp} \otimes \ldots \otimes h_M^{amp} \qquad (26)$$

Furthermore, the probability of statistic T_i^{amp} for underlying permutation test is given by

$$Pr\left(T_i^{amp}\right) = \sum_{T:T>T_i^{amp}} Pr(H^{amp}(T)) \qquad (27)$$

here $Pr(H^{amp}(T))$ is the probability under the reference histogram H^{amp} of a potential score T (also known as p-value), with larger score of T corresponding to notionally greater departure from null hypothesis. The p-values for test statistic T_i^{del} and T_i^{LOH} can also be calculated by this way. Furthermore, to produce relative

conservative result with lower Type I error rate in multiple hypothesis testing, the p-values are further corrected by using FDR procedure in TAFFYS. The corrected probability, known as q-value, is finally used to ascertain statistically significant driver aberrations.

Results

Reference

1. Wang K, Li M, Hadley D, Liu R, Glessner J, Grant SF, Hakonarson H, Bucan M: **PennCNV: an integrated hidden Markov model designed for high-resolution copy number variation detection in whole-genome SNP genotyping data**. *Genome research* 2007, **17**(11):1665-1674.
2. Peiffer DA, Le JM, Steemers FJ, Chang W, Jenniges T, Garcia F, Haden K, Li J, Shaw CA, Belmont J *et al*: **High-resolution genomic profiling of chromosomal aberrations using Infinium whole-genome genotyping**. *Genome research* 2006, **16**(9):1136-1148.
3. Nancarrow DJ, Handoko HY, Stark MS, Whiteman DC, Hayward NK: **SiDCoN: a tool to aid scoring of DNA copy number changes in SNP chip data**. *PloS one* 2007, **2**(10):e1093.
4. Hsu L, Self SG, Grove D, Randolph T, Wang K, Delrow JJ, Loo L, Porter P: **Denoising array-based comparative genomic hybridization data using wavelets**. *Biostatistics* 2005, **6**(2):211-226.
5. Li A, Liu Z, Lezon-Geyda K, Sarkar S, Lannin D, Schulz V, Krop I, Winer E, Harris L, Tuck D: **GPHMM: an integrated hidden Markov model for identification of copy number alteration and loss of heterozygosity in complex tumor samples using whole genome SNP arrays**. *Nucleic acids research* 2011, **39**(12):4928-4941.
6. Beroukhim R, Getz G, Nghiemphu L, Barretina J, Hsueh T, Linhart D, Vivanco I, Lee JC, Huang JH, Alexander S *et al*: **Assessing the significance of chromosomal aberrations in cancer: methodology and application to glioma**. *Proceedings of the National Academy of Sciences of the United States of America* 2007, **104**(50):20007-20012.
