# Supplementary material for: TAFFYS: An Integrated Tool for Comprehensive Analysis of Genomic Aberrations in Tumor Samples
Source: PLoS One. 2015 Jun 25;10(6):e0129835. doi: 10.1371/journal.pone.0129835 (PMC4482394; doi:10.1371/journal.pone.0129835)
Supplement: S2 File — (PDF) [file pone.0129835.s002.pdf]

Usage of TAFFYS

0. Introduction

TAFFYS offers an integrated analysis for genomic aberrations for Affymetrix tumor SNP array data, including signal pre-processing, aberration detection and significance test. First, Affymetrix CEL file is pre-processed to extract LRR and BAF signals. PennCNV-affy built-in module is recommended for this purpose. Then a wavelet de-noising approach is applied to suppressing noise of LRR signals. Based on the statistical distributions of the LRR and BAF signals, TAFFYS adopts a hidden Markov model (HMM) and expectation maximization (EM) algorithm for identification of genomic aberration and tumor genotypes, in which critical issues including signal variances, normal cell contamination, LRR baseline shift and GC content bias are parameterized and estimated. In addition, for multiple tumor samples, TAFFYS provides a permutation-based approach by using the absolute copy number profiles to evaluate the statistical significance of aberrations in cancer genome. TAFFYS is implemented in standalone software package, and available from the associated website: http://bioinformatics.ustc.edu.cn/taffys/.

1. Preparations

Before using TAFFYS, Affymetrix CEL files should be first converted into LRR and BAF signals. We recommend PennCNV-affy for doing this, and the details of PennCNV-affy can be found at http://www.openbioinformatics.org/penncnv/penncnv_tutorial_affy_gw6.html . It is of note that, as suggested by PennCNV-affy, if not sufficient CEL files are available, user can jump over the sub-steps 1.1 and 1.3 and alternatively use the default canonical clustering files provided in the PennCNV-affy package.

After pre-processing by PennCNV-affy, the input SNP array data file for TAFFYS should be formatted as follows:

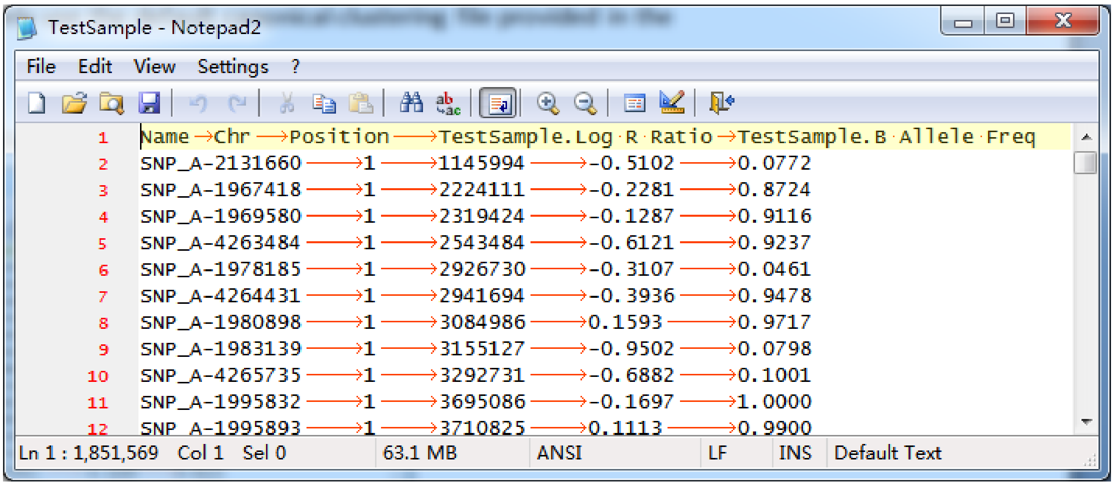

Name	Chr	Position	TestSample.Log R Ratio	TestSample.B Allele Freq
SNP_A-2131660	1	1145994	-0.5102	0.0772
SNP_A-1967418	1	2224111	-0.2281	0.8724
SNP_A-1969580	1	2319424	-0.1287	0.9116
SNP_A-4263484	1	2543484	-0.6121	0.9237
SNP_A-1978185	1	2926730	-0.3107	0.0461
SNP_A-4264431	1	2941694	-0.3936	0.9478
SNP_A-1980898	1	3084986	0.1593	0.9717
SNP_A-1983139	1	3155127	-0.9502	0.0798
SNP_A-4265735	1	3292731	-0.6882	0.1001
SNP_A-1995832	1	3695086	-0.1697	1.0000
SNP_A-1995893	1	3710825	0.1113	0.9900

In the mean time, the order of LRR and BAF signals (the 4th and 5th columns) can be switched, as shown below:

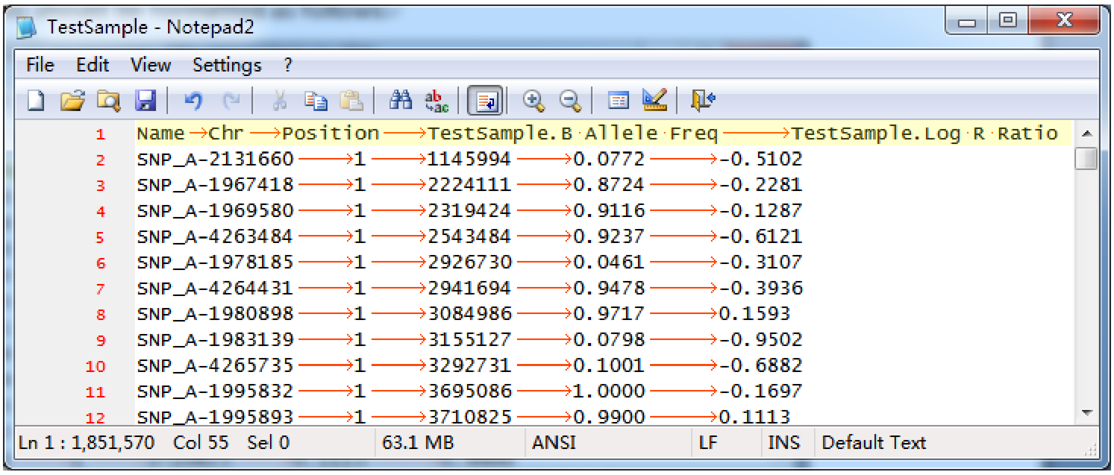

Both two formats are compatible for TAFFYS.

2. Installation

TAFFYS is implemented by Matlab/C language. To use TAFFYS, users firstly need to install MATLAB Component Runtime (MCR) library utility and the compressed file can be downloaded from http://bioinformatics.ustc.edu.cn/taffys/MCR.zip . After unzipping this file, the installation can be started with following interface:

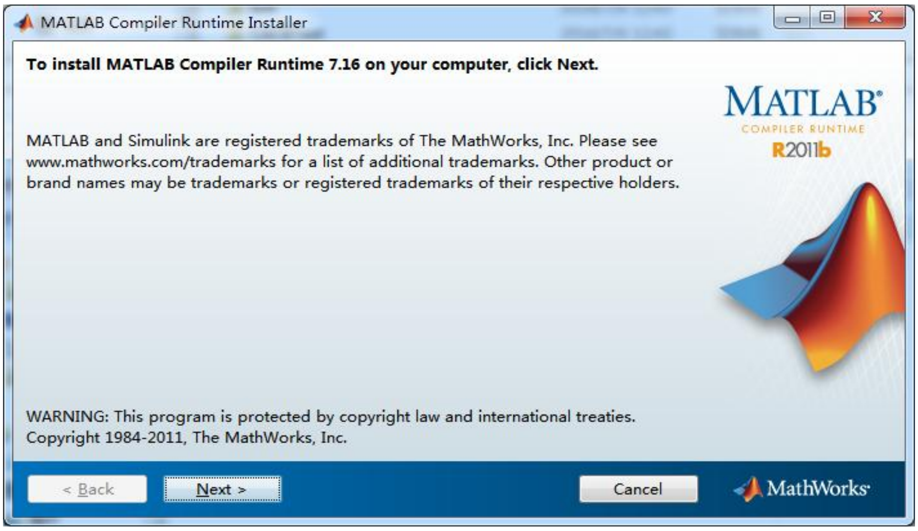

Click the “Next” all the way to finish the installation.

Next, users should download the TAFFYS program from http://bioinformatics.ustc.edu.cn/taffys/TAFFYS1.0forWin64.zip . After unzipping this file, no additional installation is needed for TAFFYS. Several files and directories are obtained for running TAFFYS

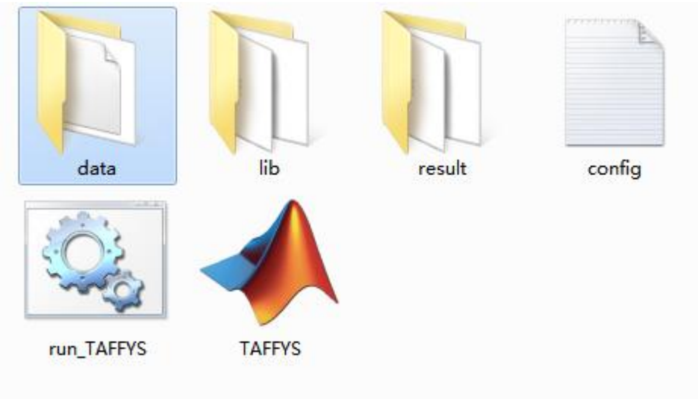

3. Usage

To run TAFFYS, users should first assign basic information for tumor SNP array data in the ‘config.txt’ file, which includes:

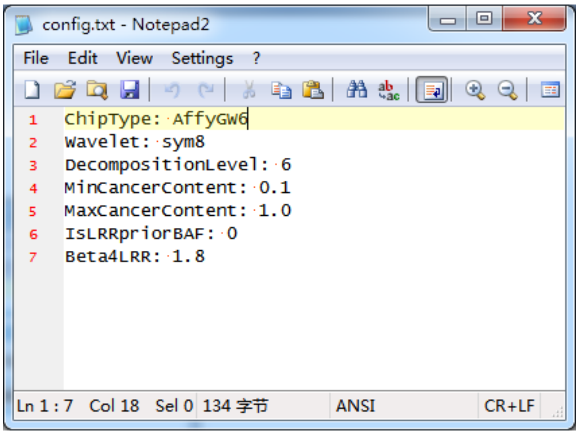

ChipType: SNP array chip information. At present, totally four types of Affymetrix SNP array are supported by TAFFYS, including “AffyGW6”, “AffyGW5”, “Affy500k” and “Affy100k” (Case Sensitive).

Wavelet: wavelet for de-noising, and user can choose “sym8” (default), “haar” and “dir” (Case Sensitive).

DecompositionLevel: the decomposition level for wavelet de-noising approach. User can assign the value from 0 to10. (0 denotes that signals will not be applied with de-noising procedure, and the default value is 6)

MinCancerContent & **MaxCancerContent**: the minimum and maximum values of the cancer cell content. The value of minimum should not be less than 0.1 and maximum should not be larger than 1.0. A narrow range of cancer cell content will assist TAFFYS and improve the aberration identification accuracy. *For example, for the cell-line sample or pure normal sample, we recommend to user assign the minimum cancer cell content as 0.95*.

IsLRRpriorBAF: the format of SNP array data file. As mentioned above, the order of LRR and BAF signals can be switched. If the 4th and 5th columns correspond to LRR and BAF signals respectively, then **IsLRRpriorBAF** = 1. Otherwise, **IsLRRpriorBAF** = 0, if the 4th and 5th

columns correspond to BAF and LRR signals, respectively.
Beta4LRR: contraction coefficient for LRR signal, and details see article.

#: It should be noted that if users are not sure about the input information about these items, please use the default values instead.

After inputting all required information, users can execute TAFFYS with following command:

TAFFYS.exe ./data ./result ./config.txt

The 2nd argument assigns the SNP array data directory, the 3rd argument assigns the result directory and the 4th argument assigns the configuration file. Besides, this command is also written into a file named “run_TAFFYS”, users can run TAFFYS program by using this file.

For Windows user, click ‘Start’ -> ‘run’ -> ‘cmd’. The interface is shown as follows:

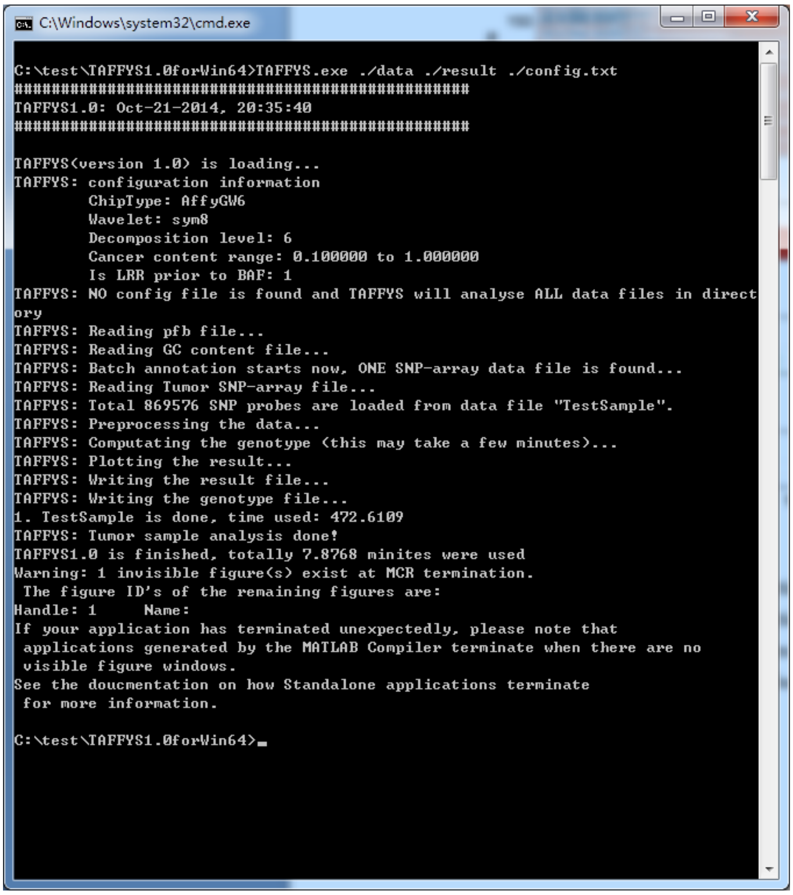

For Linux user, the command can be executed in Terminal.

4. Output

There are totally five types of output file generated by TAFFYS, including result file, genotype file, batch summary information file, significance test result file and visualization.

Result file

The aberration results are saved in a text format file named as “*TumorFileName.result*”. This file contains two sections: tumor sample parameters and segmented genomic copy number profiles. For tumor sample parameter section, the data is formatted as:

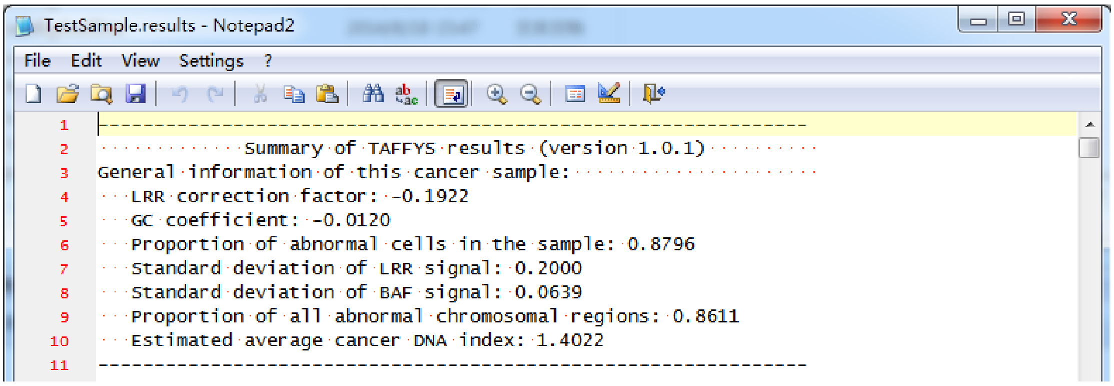

```
TestSample.results - Notepad2
-----------------------------------------------------------------
             Summary of TAFFYS results (version 1.0.1)
General information of this cancer sample:
   LRR correction factor: -0.1922
   GC coefficient: -0.0120
   Proportion of abnormal cells in the sample: 0.8796
   Standard deviation of LRR signal: 0.2000
   Standard deviation of BAF signal: 0.0639
   Proportion of all abnormal chromosomal regions: 0.8611
   Estimated average cancer DNA index: 1.4022
-----------------------------------------------------------------
```

For the segmented genomic copy number profile section, the data is formatted as:

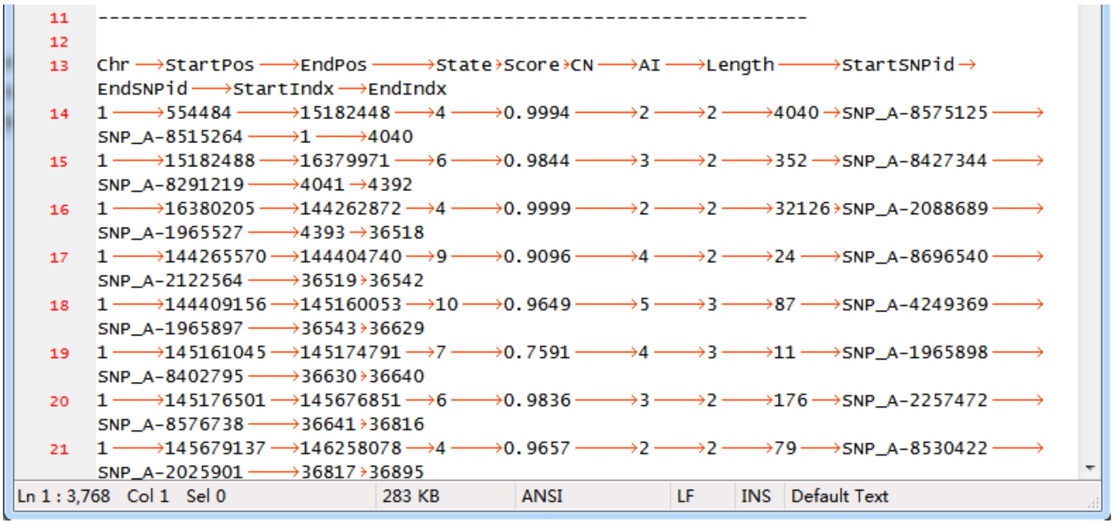

```
-----------------------------------------------------------------

Chr	StartPos	EndPos	State	Score	CN	AI	Length	StartSNPid	EndSNPid	StartIndx	EndIndx
1	554484	15182448	4	0.9994	2	2	4040	SNP_A-8575125	SNP_A-8515264	1	4040
1	15182488	16379971	6	0.9844	3	2	352	SNP_A-8427344	SNP_A-8291219	4041	4392
1	16380205	144262872	4	0.9999	2	2	32126	SNP_A-2088689	SNP_A-1965527	4393	36518
1	144265570	144404740	9	0.9096	4	2	24	SNP_A-8696540	SNP_A-2122564	36519	36542
1	144409156	145160053	10	0.9649	5	3	87	SNP_A-4249369	SNP_A-1965897	36543	36629
1	145161045	145174791	7	0.7591	4	3	11	SNP_A-1965898	SNP_A-8402795	36630	36640
1	145176501	145676851	6	0.9836	3	2	176	SNP_A-2257472	SNP_A-8576738	36641	36816
1	145679137	146258078	4	0.9657	2	2	79	SNP_A-8530422	SNP_A-2025901	36817	36895
Ln 1 : 3,768   Col 1   Sel 0   283 KB   ANSI   LF   INS   Default Text
```

Chr: Chromosome.

StartPos: The start position of each aberration region.

EndPos: The end position of each aberration region.

State: The aberration state in HMM, which is defined in the article of TAFFYS (Table S1).

Score: The goodness score for observed signals under the given state, which can be used to reflect the discrepancy between observed and the expected values.

CN: Copy number of each aberration region.

AI: Allelic imbalance status of current segment (1: deletion; 2: LOH; 3: heterozygosity)

Length: Length of current segment.

StartSNPid: The ID of the first SNP in current segment.

EndSNPid: The ID of the last SNP in current segment.

StartIndx: The index of the first SNP in current segment.

EndIndx: The index of the last SNP in current segment .

Genotype file

The genotype file is saved in the result directory with suffix as .Gtype. Gtype file provides

genotypes of each probe in tumor sample, and is formatted as:

TestSample.Gtype - Notepad2

Name	Chr	position	CN	B allele CN	State	Genotype
SNP_A-8575125	1	554484	2	0	4	AA
SNP_A-8575371	1	557616	2	2	4	BB
SNP_A-8709646	1	711153	2	0	4	AA
SNP_A-8497791	1	730720	2	0	4	AA
SNP_A-1909444	1	742429	2	2	4	BB
SNP_A-8358063	1	751595	2	2	4	BB
SNP_A-8329892	1	755132	2	0	4	AA
SNP_A-8408912	1	766985	2	2	4	BB
SNP_A-1886933	1	775852	2	2	4	BB
SNP_A-2236359	1	782343	2	2	4	BB
SNP_A-8515688	1	788822	2	2	4	BB
SNP_A-2205441	1	789326	2	0	4	AA
SNP_A-8524447	1	878522	2	0	4	AA
SNP_A-8573955	1	908436	2	2	4	BB
SNP_A-8530278	1	916294	2	2	4	BB
SNP_A-8573668	1	936897	2	2	4	BB
SNP_A-8573414	1	939471	2	2	4	BB
SNP_A-8531044	1	980280	2	0	4	AA

Ln 1 : 869,578 Col 1 Sel 0 29.4 MB ANSI LF INS Default Text

Name: the SNP name (rsID);
Chr: Chromosome.
Position: The position of each SNP.
CN: Copy number of each SNP.
B allele CN: Copy number of B allele for each SNP.
State: The aberration state in HMM, which is defined in the article of TAFFYS (Table S1).
Genotype: The identified genotype of each SNP.

Batch summary

Two summary files (“Tumor_Summary.txt” & “Tumor_Summary4LowQualitySample.txt”) provide the briefly information of all analyzed samples, including TAFFYS version, time stamp, and important parameters collected from each result report file. The data in this file is formatted as follows:

TumorSummary.txt - Notepad2

Version:	Date	Time	Sample	Tumor content	Baseline shift	GC coef.	ACN	BAF_Het Sigma	BAF_Homo Sigma	LRR sigma	K
TAFFYS1.0	Oct-21-2014	20:42:17	TestSample	0.946573	-0.181705	-0.010673	2.814945	0.053059	0.093633	0.100000	-0.357900

Ln 1 : 3 Col 1 Sel 0 226 字节 ANSI LF INS Default Text

It is of note that the results of sample with very large signal deviations, GC coefficient and high average copy number will be saved in “Tumor_Summary4LowQualitySample.txt”, and not be used in further significance test.

Significance test result

This file (.sig file) gives the results of significance test for genomic aberrations. It only will be performed after analyzing multiple tumor samples, since too few (<=4) samples cannot be used to precisely calculate the statistical significance. Three kinds of logarithmic scaled q-values (representing amplification, deletion and LOH) are provided in this file, and the higher absolute value notionally implies higher statistical significance of corresponding aberration region. The data in this file is formatted as follows:

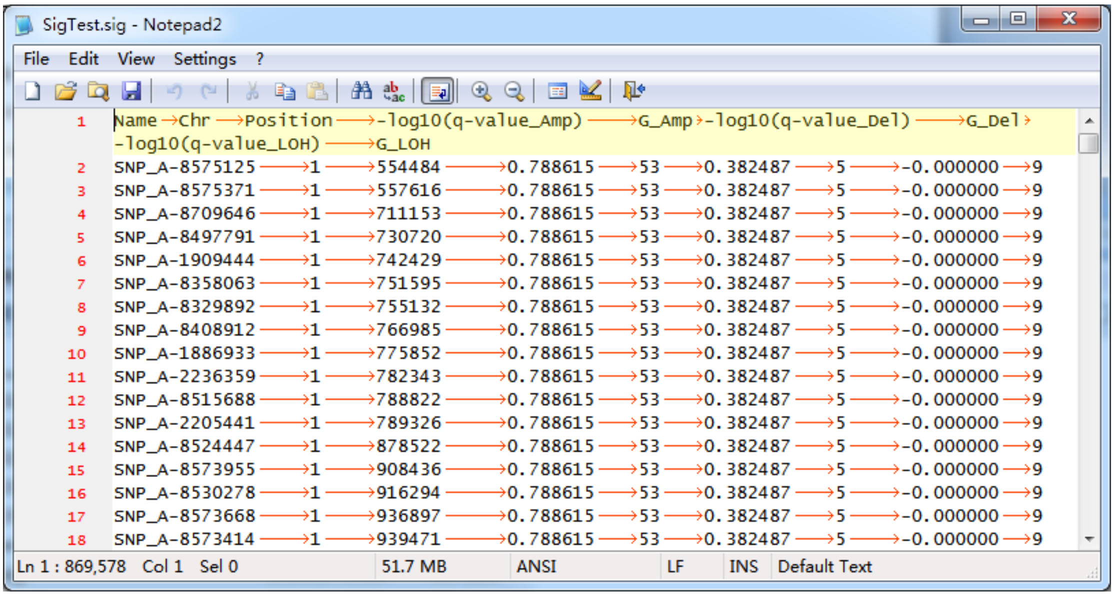

Name	Chr	Position	-log10(q-value_Amp)	G_Amp	-log10(q-value_Del)	G_Del	-log10(q-value_LOH)	G_LOH
SNP_A-8575125	1	554484	0.788615	53	0.382487	5	-0.000000	9
SNP_A-8575371	1	557616	0.788615	53	0.382487	5	-0.000000	9
SNP_A-8709646	1	711153	0.788615	53	0.382487	5	-0.000000	9
SNP_A-8497791	1	730720	0.788615	53	0.382487	5	-0.000000	9
SNP_A-1909444	1	742429	0.788615	53	0.382487	5	-0.000000	9
SNP_A-8358063	1	751595	0.788615	53	0.382487	5	-0.000000	9
SNP_A-8329892	1	755132	0.788615	53	0.382487	5	-0.000000	9
SNP_A-8408912	1	766985	0.788615	53	0.382487	5	-0.000000	9
SNP_A-1886933	1	775852	0.788615	53	0.382487	5	-0.000000	9
SNP_A-2236359	1	782343	0.788615	53	0.382487	5	-0.000000	9
SNP_A-8515688	1	788822	0.788615	53	0.382487	5	-0.000000	9
SNP_A-2205441	1	789326	0.788615	53	0.382487	5	-0.000000	9
SNP_A-8524447	1	878522	0.788615	53	0.382487	5	-0.000000	9
SNP_A-8573955	1	908436	0.788615	53	0.382487	5	-0.000000	9
SNP_A-8530278	1	916294	0.788615	53	0.382487	5	-0.000000	9
SNP_A-8573668	1	936897	0.788615	53	0.382487	5	-0.000000	9
SNP_A-8573414	1	939471	0.788615	53	0.382487	5	-0.000000	9

Name: the SNP name (rsID);

Chr: Chromosome.

Position: The position of each SNP.

-log10(q-value_xxx): The logarithmic scaled Q-value of xxx(amplification, deletion, and LOH) aberration for each SNP, details sees the Methods of TAFFYS.

G_xxx: The G score of xxx(amplification, deletion, and LOH) aberration for each SNP, details sees the Methods of TAFFYS.

Visualization

In the end of analysis, TAFFYS will also generate the visualization of identification result for each chromosome. An example of the visualization is shown as follows:

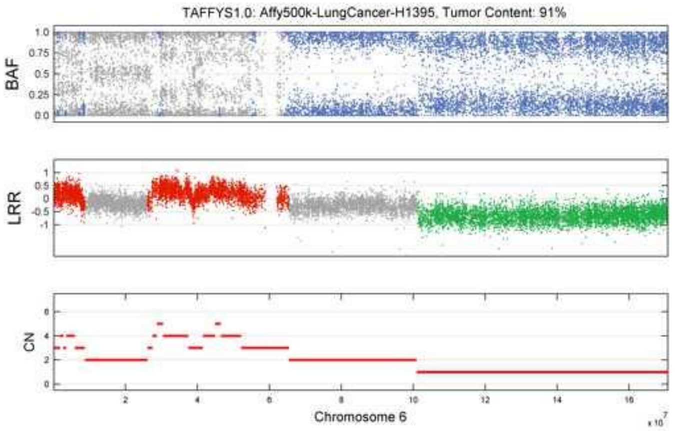

Each chromosome consists of three subplots. First subplot (top subplot) shows BAF signals and based on the aberration type, the LOH regions are marked with blue color. The second subplot shows LRR signals, and four colors: deep green, green, gray and red denote the homozygous deletion, hemizygous deletion, copy neutral and amplification, respectively. The bottom subplot illustrates the copy number profile.

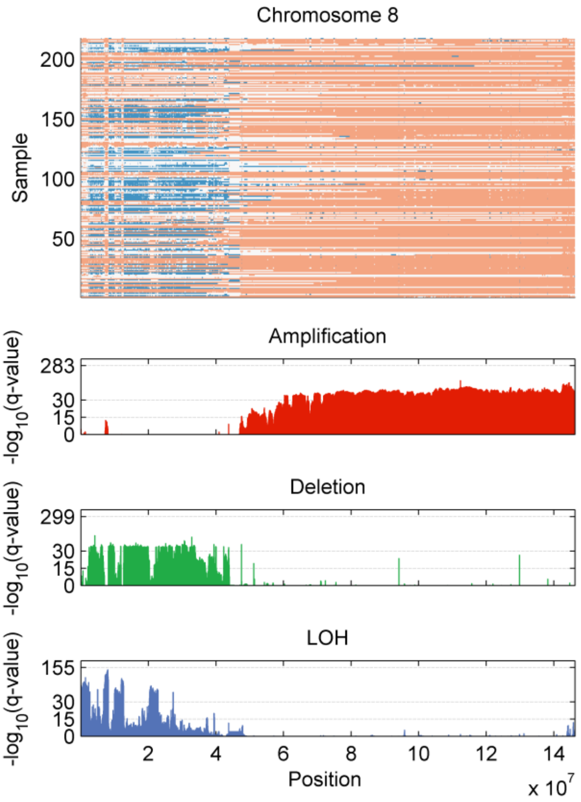

The visualization of significance test is also generated for each chromosome. The top subplot illustrates amplification (orange) /deletion (blue) profile on corresponding chromosome, and visualized q-values for amplification, deletion and LOH generated from significance test are shown in three subplots below.

5. Quick example

Data files
For a quick start, we provide a tumor sample for test, and this data file is packed in compressed TAFFYS program file (also see above section 2 **Installation**; URL: http://bioinformatics.ustc.edu.cn/taffys/TAFFYS1.0forWin64.zip). In directory *data*, user can see the test data file named "*TestSample*".
Run TAFFYS
To run this data, user can simply double click "*run_TAFFYS.bat*" in parent directory in Windows OS. (For Linux user, run the "*run_TAFFYS.sh*" in Terminal.)

6. Dataset in TAFFYS

In article of TAFFYS, there are three dataset used for performance evaluation:

1. Dilution series dataset of lung tumor (Affymetrix GenomeWide SNP 6.0)
   This dataset contains four tumor samples, with cancer cell proportions of 30%, 50%, 70% and 100%. The original data files are downloaded from GEO website, with accession number GSE29172 . (http://www.ncbi.nlm.nih.gov/geo/query/acc.cgi?acc=GSE29172). Besides, we provide the LRR/BAF signal files pre-processed by PennCNV-affy (http://bioinformatics.ustc.edu.cn/taffys/GSE29172FromPennCNVaffy.zip). The results of these samples from TAFFYS can also be downloaded from (http://bioinformatics.ustc.edu.cn/taffys/ResultsOfGSE29172FromTAFFYS.zip).
2. Breast cancer sample 7204 (Affymetrix GenomeWide SNP 5.0 & Illumina HumanCNV370k)
   This sample is both analyzed by Affymetrix GenomeWide SNP 5.0 & Illumina HumanCNV370k, and original data files are downloaded from GEO website, with accession number GSE16400 (http://www.ncbi.nlm.nih.gov/geo/query/acc.cgi?acc=GSE164000). The LRR/BAF signal files pre-processed by PennCNV-affy can be downloaded from TAFFYS website (http://bioinformatics.ustc.edu.cn/taffys/GSE16400FromPennCNVaffy.zip). The result files of this sample from TAFFYS can also be downloaded from (http://bioinformatics.ustc.edu.cn/taffys/ResultsOfGSE16400FromTAFFYS.zip).
3. Pure lung tumor (Affymetrix Mapping 500k)
   This sample is the same with lung tumor sample in dataset 1, with only difference in analysis platform (Affymetrix Mapping 500k). The original data files are downloaded from GEO website, with accession number GSE17247. (http://www.ncbi.nlm.nih.gov/geo/query/acc.cgi?acc=GSE17247). The LRR/BAF signal file pre-processed by PennCNV-affy can be downloaded from (http://bioinformatics.ustc.edu.cn/taffys/GSE17247FromPennCNVaffy.zip). The results of this data file from TAFFYS can also be downloaded from (http://bioinformatics.ustc.edu.cn/taffys/ResultsOfGSE17247FromTAFFYS.zip).
