# Supplementary figures and images for: TAFFYS: An Integrated Tool for Comprehensive Analysis of Genomic Aberrations in Tumor Samples
Source: PLoS One. 2015 Jun 25;10(6):e0129835. doi: 10.1371/journal.pone.0129835 (PMC4482394; doi:10.1371/journal.pone.0129835)

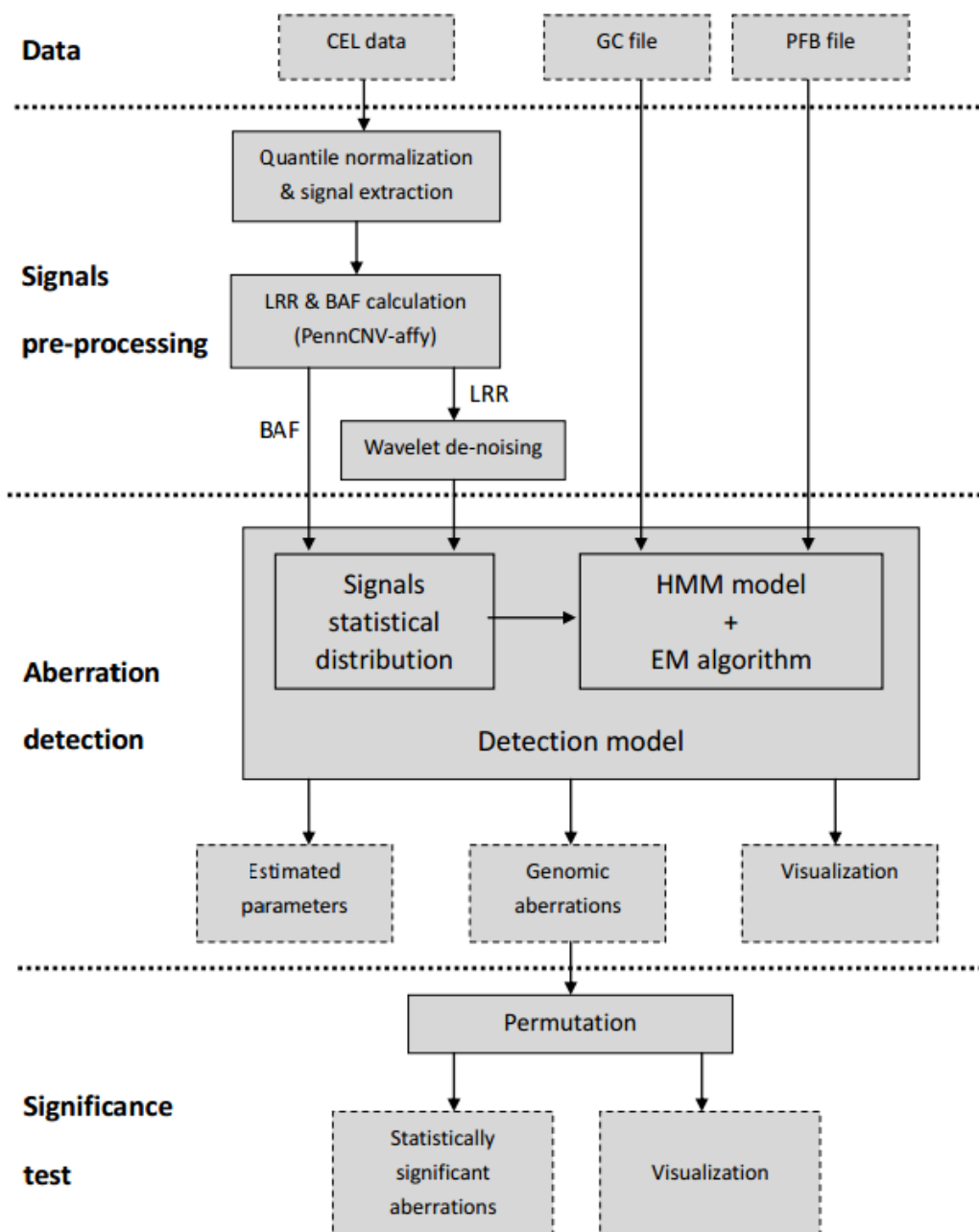

Figure S1 The entire pipeline of TAFFYS.

Supplement: S1 Fig — (PDF) [file pone.0129835.s003.pdf]
