# Supplementary material for: TAFFYS: An Integrated Tool for Comprehensive Analysis of Genomic Aberrations in Tumor Samples
Source: PLoS One. 2015 Jun 25;10(6):e0129835. doi: 10.1371/journal.pone.0129835 (PMC4482394; doi:10.1371/journal.pone.0129835)
Supplement: S2 Fig — (a) Results of processed LRR signals at different wavelet decomposition levels, including 2, 3, 5 and 6. (b) Illustration of corresponding LRR variances at different decomposition levels. (PDF) [file pone.0129835.s004.pdf]

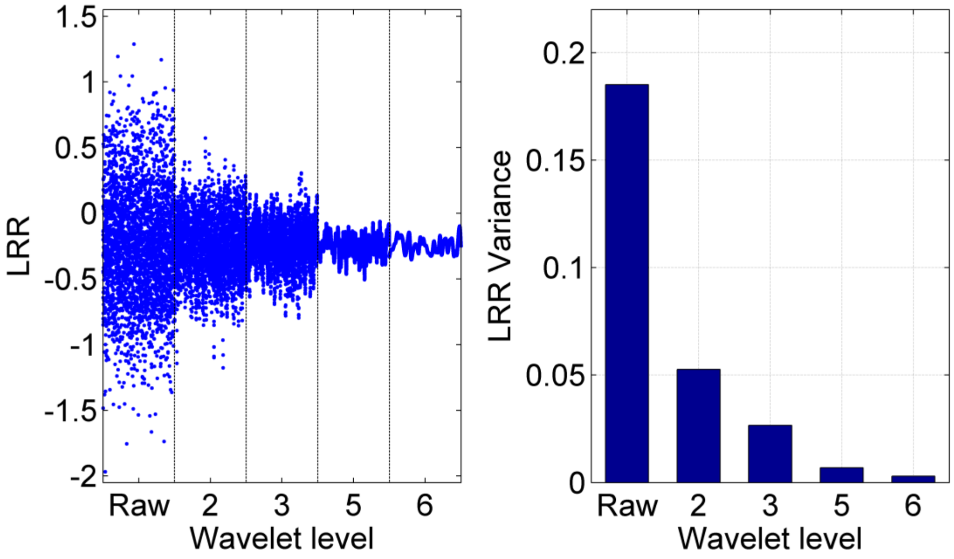

Figure S2 **Performance evaluation of wavelet de-noising on LRR signals.** (a) Results of processed LRR signals at different wavelet decomposition levels, including 2, 3, 5 and 6. (B) Illustration of corresponding LRR variances at different decomposition levels.
