# Supplementary material for: TAFFYS: An Integrated Tool for Comprehensive Analysis of Genomic Aberrations in Tumor Samples
Source: PLoS One. 2015 Jun 25;10(6):e0129835. doi: 10.1371/journal.pone.0129835 (PMC4482394; doi:10.1371/journal.pone.0129835)
Supplement: S3 Fig — Genome-wide amplification (red) /deletion (blue) profiles for dilution samples with cancer cell content ranging from 30% to 100%. (PDF) [file pone.0129835.s005.pdf]

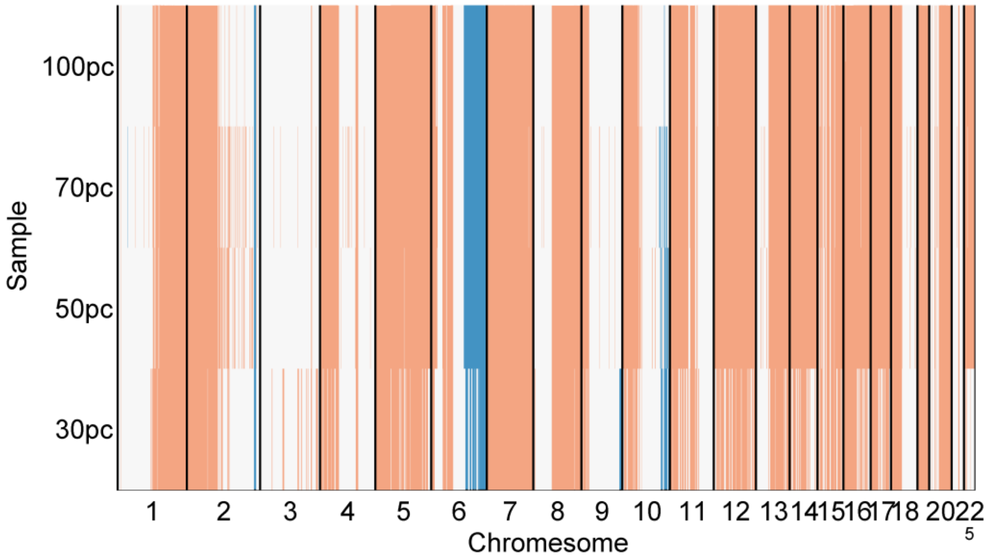

Figure S3. **Assessment of genomic aberration identification for TAFFYS using dilution series data.** Genome-wide amplification (red) /deletion (blue) profiles for dilution samples with cancer cell content ranging from 30% to 100%.
