# Supplementary material for: TAFFYS: An Integrated Tool for Comprehensive Analysis of Genomic Aberrations in Tumor Samples
Source: PLoS One. 2015 Jun 25;10(6):e0129835. doi: 10.1371/journal.pone.0129835 (PMC4482394; doi:10.1371/journal.pone.0129835)
Supplement: S4 Fig — The results of genome-wide aberration identification on chromosome 10 using lung cancer H1395. For the BAF panel, LOH region is marked with blue, while non-LOH region with gray. For LRR panel, amplification is colored with red, and deletion with green. Black dots denote the signals after performing de-noising. For copy number (CN) panel, red line correspond to the copy number, and blue dots denote the goodness scores, which are only plotted when they are smaller than 0.05. (PDF) [file pone.0129835.s006.pdf]

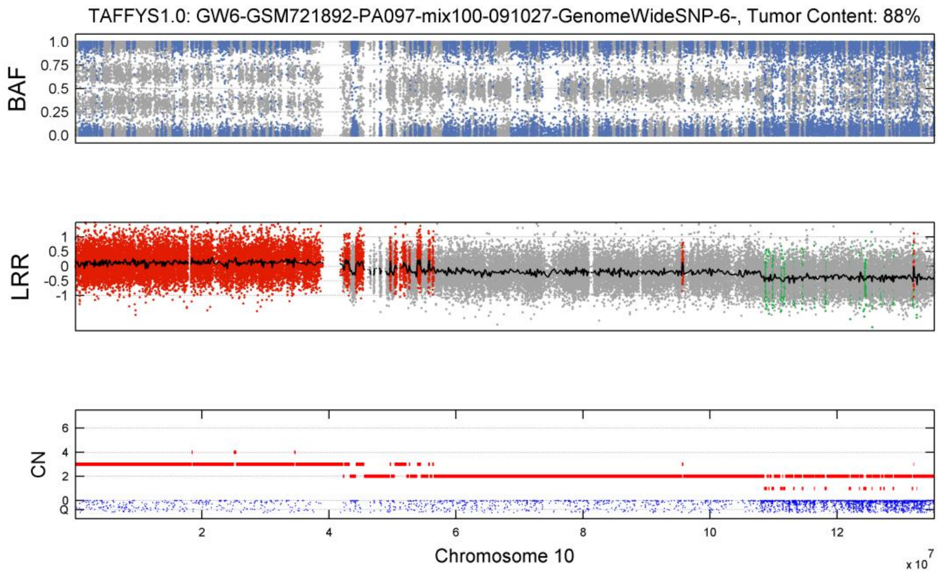

Figure S4. **Aberration identification of TAFFYS using lung cancer H1395.** The results of genome-wide aberration identification on chromosome 10 using lung cancer H1395. For the BAF panel, LOH region is marked with blue, while non-LOH region with gray. For LRR panel, amplification is colored with red, and deletion with green. Black dots denote the signals after performing de-noising. For copy number (CN) panel, red line correspond to the copy number, and blue dots denote the goodness scores, which are only plotted when they are smaller than 0.05.
