# Supplementary material for: TAFFYS: An Integrated Tool for Comprehensive Analysis of Genomic Aberrations in Tumor Samples
Source: PLoS One. 2015 Jun 25;10(6):e0129835. doi: 10.1371/journal.pone.0129835 (PMC4482394; doi:10.1371/journal.pone.0129835)
Supplement: S5 Fig — The results of genome-wide aberration identification using lung cancer H1395, which are analyzed by TAFFYS using Affymetrix GenomeWideSNP6.0 (bottom) and Affymetrix Mapping 500k (top). (PDF) [file pone.0129835.s007.pdf]

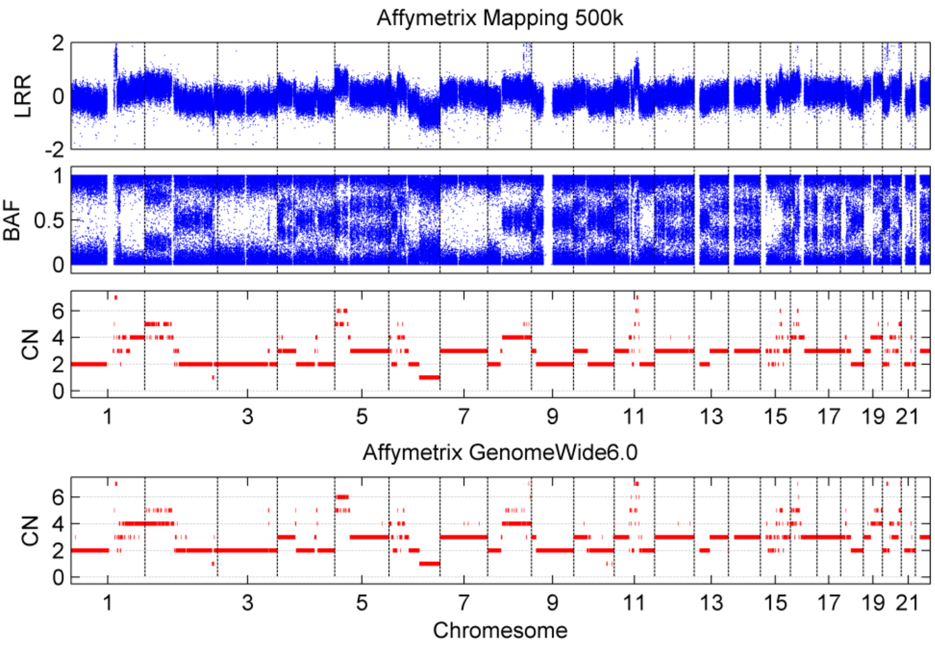

Figure S5. **Comparison of genomic aberration identification of TAFFYS between GenomeWideSNP6.0 and Mapping500k.** The results of genome-wide aberration identification using lung cancer H1395, which are analyzed by TAFFYS using Affymetrix GenomeWideSNP6.0 (bottom) and Affymetrix Mapping 500k (top).
