# Supplementary material for: TAFFYS: An Integrated Tool for Comprehensive Analysis of Genomic Aberrations in Tumor Samples
Source: PLoS One. 2015 Jun 25;10(6):e0129835. doi: 10.1371/journal.pone.0129835 (PMC4482394; doi:10.1371/journal.pone.0129835)
Supplement: S1 Table — (PDF) [file pone.0129835.s008.pdf]

Table S1 Detailed information of hidden states in TAFFYS

State (s)	Genotypes (BAF>=0.5)	$(m_{i,t}, n_{i,t})$*	Description
1	N/A	(0,0)	Deletion of two copies
2	'B'	(0,1)	Deletion of one copy
3	'AB';'BB'	(1,2)	Normal
4	'BB'	(2,2)	Copy neutral with LOH
5	'ABB';'BBB'	(2,3)	Three copies with duplication of one allele
6	'BBB'	(3,3)	Three copies with LOH
7	'ABBB';'BBBB'	(3,4)	Four copies with duplication of one allele
8	''AABB';'BBBB'	(2,4)	Four copies with duplication of both alleles
9	'BBBB'	(4,4)	Four copies with LOH
10	'ABBBB';'BBBBB'	(4,5)	Five copies with duplication of one allele
11	'AABBB';'BBBBB'	(3,5)	Five copies with duplication of both alleles
12	'BBBBB'	(5,5)	Five copies with LOH
13	'ABBBBB';'BBBBBB'	(5,6)	Six copies with duplication of one alleles
14	'AABBBB';'BBBBBB'	(4,6)	Six copies with duplication of both alleles
15	''AAABBB';'BBBBBB'	(3,6)	Six copies with duplication of both alleles
16	'BBBBBB'	(6,6)	Six copies with LOH
17	'ABBBBBB';'BBBBBBB'	(6,7)	Seven copies with duplication of one alleles
18	'AABBBBB';'BBBBBBB'	(5,7)	Seven copies with duplication of both alleles
19	' AAABBBB';'BBBBBBB'	(4,7)	Seven copies with duplication of both alleles
20	'BBBBBBB'	(7,7)	Seven copies with LOH

*Only genotypes associated with heterozygous normal genotype 'AB' are shown.
