# Supplementary material for: TAFFYS: An Integrated Tool for Comprehensive Analysis of Genomic Aberrations in Tumor Samples
Source: PLoS One. 2015 Jun 25;10(6):e0129835. doi: 10.1371/journal.pone.0129835 (PMC4482394; doi:10.1371/journal.pone.0129835)
Supplement: S2 Table — (PDF) [file pone.0129835.s009.pdf]

Table S2 Detailed information of eliminated samples

Sample	GC coef.	BAF_Het Sigma	BAF_Homo Sigma	Description
GSM805452	0.15	0.09	0.08	High GC bias
GSM805454	0.14	0.05	0.08	High GC bias
GSM805456	-0.01	0.10	0.04	High signal noise
GSM805460	0.09	0.10	0.06	High GC bias
GSM805464	0.04	0.13	0.07	High signal noise
GSM805479	0.21	0.12	0.12	High GC bias
GSM805491	0.03	0.12	0.07	High signal noise
GSM805492	0.11	0.05	0.11	High GC bias
GSM805493	0.05	0.12	0.07	High signal noise
